# Diet, Activity and Sleep Clusters Associated With Obesity Markers of Children in the US‐Affiliated Pacific

**DOI:** 10.1111/apa.70012

**Published:** 2025-02-24

**Authors:** Dorothea Dumuid, Ashley B. Yamanaka, Kar Hau Chong, Anthony D. Okely, Lynne R. Wilkens, Yurii B. Shvetsov, Chloe P. Lozano, Rachel Novotny

**Affiliations:** ^1^ Alliance for Research in Exercise, Nutrition and Activity, Allied Health & Human Performance University of South Australia Adelaide South Australia Australia; ^2^ College of Tropical Agriculture and Human Resilience University of Hawaii at Manoa Honolulu Hawaii USA; ^3^ School of Social Sciences, Early Start, Faculty of the Arts, Social Sciences and Humanities University of Wollongong Wollongong New South Wales Australia; ^4^ Cancer Center University of Hawaii at Manoa Honolulu Hawaii USA

**Keywords:** energy balance, macronutrient composition, movement behaviour, physical activity, screen time, sleep

## Abstract

**Aim:**

Among children in the US‐Affiliated Pacific, we aimed to identify lifestyle clusters and associations with obesity.

**Methods:**

Movement behaviours, diet and anthropometrics were from the Children's Healthy Living Program (*n* = 1780; 2012–2015). Partitioning‐around‐medoids identified clusters; regression examined differences in anthropometrics.

**Results:**

Among 2–5‐year‐olds, boys' clusters were: (1) high %energy from (*E*%) fat and sedentary behaviour; (2) high screen time and energy intake and (3) long sleep. Body mass index z‐score (zBMI) was lower in Cluster 3 versus 1 (−0.28 [−0.50; −0.07], *p* = 0.01). Girls' clusters were: (1) high energy intake; (2) low *E*% fat and (3) high physical activity and sleep. zBMI was lower in Cluster 3 versus 1 (−0.34 [−0.55; −0.13], *p* = 0.002). Among 6–8‐year‐olds, boys' clusters were: (1) high screen time; (2) high energy intake; (3) high *E*% protein; (4) long sleep and (5) high sedentary time and low *E*% saturated fat. Compared with Cluster 1, zBMI was lower in Clusters 3 (−0.43 [−0.84; −0.02], *p* = 0.04), 4 (−0.64 [−1.08; −0.20], *p* = 0.004) and 5 (−0.93 [−1.35; −0.51], *p* < 0.001). Girls' clusters were: (1) high *E*% fat and protein; (2) high screen time and energy intake; (3) short sleep and high physical activity (4) long sleep and (5) low sedentary time. Compared with Cluster 1, zBMI was lower in Clusters 2 (−0.57 [−0.98; −0.17], *p* = 0.006), 3 (−0.48 [−0.81; −0.14], *p* = 0.005) and 5 (−0.50 [−0.83; −0.18], *p* = 0.003).

**Conclusion:**

Various lifestyle patterns support healthy body weight.

**Trial Registration:**

ClinicalTrials.gov identifier: NCT01881373

**Abbreviations**
CHL: Children's Healthy Living
CNMI: Commonwealth of the Northern Mariana Islands
FAS: freely associated states
LPA: light‐intensity physical activity
MVPA: moderate‐to vigorous‐intensity physical activity
PAM: partitioning around medoids
US: United States
USA: United States of America
zBMI: body mass index *z*‐score

**Summary**

- Children's lifestyle behaviours influence risk of obesity, yet this relationship remains under explored among children in the US‐Affiliated Pacific.
- Clusters of diet, activity and sleep behaviours were associated with obesity markers in different ways for boys and girls, yet high screen time or sedentary behaviour, % energy from fat or total energy intake were consistently unfavourably associated.
- A range of lifestyle improvements across diet, activity and sleep behaviours could support healthy body weight.

## 1 Introduction

Obesity is a significant risk to children's present and future health. Escalating rates of obesity are present in the youngest populations, including early childhood [1]. Disparities exist across world regions, with the most recent (2022) estimates placing Polynesian and Micronesian countries among those with the highest rates of childhood obesity globally [2]. While it is widely accepted that lifestyle behaviours, specifically the imbalance between energy intake and energy expenditure, lead to excessive body weight, these relationships have rarely been explored among young children and even less so among young children from Polynesian and Micronesian communities in the north Pacific.

Lifestyle behaviours that are important to the balance of energy include dietary intake of energy from macronutrients (protein, carbohydrates and fats) and time spent in physical activity, sedentary behaviour and sleep [3]. The relationships between each of these behaviours, or groups of these behaviours, and adiposity have been explored previously in many populations [4]. Dietary behaviours that have been consistently associated with higher levels of obesity include excessive energy intake, a high intake of fats (particularly saturated fats), high intake of some types of carbohydrates (starch, sucrose and high fructose corn syrup) and either an insufficient or excessive intake of protein [5]. Higher levels of obesity have also been associated with low levels of physical activity, high amounts of sedentary screen time and insufficient or excessive sleep duration [6].

However, these lifestyle behaviours do not occur in isolation. In real life, these behaviours co‐occur together in lifestyle patterns. Previous studies of lifestyle behaviour clusters in child (school‐aged) populations have found similar lifestyle behaviour profiles among children from different countries across diverse socioeconomic and geographic backgrounds [7]. In both boys and girls, lifestyle behaviour profiles have been associated with varying adiposity outcomes. For example, the ‘Sitters’ profile, i.e., high sedentary time and low physical activity, has been linked with higher body fat percent, body mass index (BMI), waist‐to‐height ratio and risk of overweight and obesity compared to other lifestyle behaviour profiles [8]. However, lifestyle patterns and their associations with markers of obesity have not yet been explored among children from Polynesian and Micronesian communities of the Pacific, even though these children are among those at highest risk of obesity [2]. For this reason, it is important to characterise children's lifestyle behaviours and understand how lifestyle patterns are associated with markers of obesity among this understudied population of young people.

We aimed to explore the lifestyle profiles of children (aged 2–8 years) from Polynesian and Micronesian states in the United States‐Affiliated Pacific region, by determining how children cluster together based on their diet (total energy intake and composition of macronutrients expressed as percent of energy from macronutrients) and movement (physical activity, sedentary behaviours and sleep) behaviours. We also aimed to investigate whether BMI and waist‐to‐height ratio differed among the lifestyle behaviour clusters.

## 2 Methods

### 2.1 Study Design and Participants

The Children's Healthy Living (CHL) program is a multi‐jurisdictional prevalence study and intervention trial for children 2–8 years old in the United States‐Affiliated Pacific region [9]. Participants were recruited from 24 selected communities across 11 jurisdictions (Hawai‘i, Alaska, Commonwealth of the Northern Mariana Islands, Guam, American Samoa, Palau, Republic of Marshall Islands and the four Federated States of Micronesia: Pohnpei, Yap, Kosrae and Chuuk) in a community cluster design. The Federated States of Micronesia, Palau and Republic of Marshall Islands are treated as a group and called the Freely Associated States. This study uses cross‐sectional data, which includes the prevalence study data from the Freely Associated States and prevalence (i.e., baseline intervention) study data from the other jurisdictions. Between 2012 and 2014, a total of 5775 children participated in the CHL prevalence study, with a pre‐selected subsample of 3529 children who completed 2‐day food records (Figure S1). Institutional review board approval was obtained from the University of Hawaii at Manoa, University of Guam and University of Alaska. The Northern Marianas College, American Samoa Community College and FAS ceded approval to the University of Hawaii at Manoa. Written informed consent and assent were obtained from the caregiver and assent from the child participant.

### 2.2 Measures

#### 2.2.1 Macronutrient Composition and Total Dietary Energy Intake

Dietary records were collected from 3529 children in the CHL program, completed by a caregiver as previously described [10]. Briefly, the caregivers were provided a paper log and asked to record description, time and portion size for all foods and beverages consumed during two non‐consecutive 24‐h periods. Two days were randomly assigned for food records to ensure representation of all days of the week across children. The information for all reported foods and beverages was entered and analysed using PacTrac software [11] (with a database containing foods of the region) to determine daily intake of nutrients and other dietary components. Of interest for this analysis were daily grams of saturated fats, unsaturated fats (calculated by subtracting saturated fats from total fats), protein, added sugar and other carbohydrates (calculated by subtracting added sugar from total carbohydrates). Added sugar was computed based on published methods [12]. The daily amounts were then converted to units of energy (kcal) by multiplying grams of fats by 9 and grams of protein and carbohydrates by 4. Finally, to represent macronutrient composition, these variables were expressed as percentages of total energy intake (calculated as the sum of energy intake of all five diet variables). The dietary intake components were averaged across the 2 days per child, weighted for weekday and weekend days [13].

#### 2.2.2 24‐h Movement Behaviour Composition

Time spent in sleep, sedentary time and physical activity was assessed using an Actical accelerometer (Z series, Phillips Respironics Inc.; Murrysville, Pennsylvania) worn on the non‐dominant wrist for seven consecutive days. The Actical accelerometer was selected based on good agreement between the devices and observation in a pilot test of the devices in young children in Year 1 of CHL study [14]. The accelerometers were initialized to capture data in 1‐s epochs [9]. Data were re‐integrated into 15‐s epochs and exported as .dat files for cleaning and processing using an automated script developed in MATLAB (MathWorks, Natick, Massachusetts, USA). Briefly, the automated script included a count‐scaled algorithm to estimate sleep onset (i.e., start of first 15 continuous minutes of sleep preceded by 5 min of awake time) and sleep offset (i.e., last of 15 continuous minutes of sleep followed by 5 min of awake time) for each individual day [15]. The algorithm detects sleep and wake times using a ‘time flag’ of 8:00 PM and 7:00 AM, respectively. These times were chosen based on guidance from local researchers regarding the earliest likely bedtime and the latest likely wake time for children of this age group [16]. Using gold‐standard polysomnography as the criterion method, the count‐scaled algorithm applied to Actical wrist‐worn accelerometry data demonstrated moderate agreement (prevalence‐adjusted bias‐adjusted Kappa statistic = 0.79) in identifying sleep and wake episodes among children aged 5–8 years based on a 15‐s epoch‐by‐epoch comparison [15]. The algorithm showed high accuracy (86.0%) and sensitivity (98.0%), but low specificity (45.7%), indicating a tendency to misclassify wake periods as sleep. Despite this, the algorithm provided comparable estimates of sleep duration, with no statistically significant difference observed in sleep period time window length (Actical minus polysomnography: −15 min [−32, 2]) or total sleep duration (Actical minus polysomnography: 9 min [−14, 32]) when compared to polysomnography [15]. After excluding the non‐wear period(s) (defined as at least 20 min of consecutive zero counts) [17], the awake time data were categorised in 15‐s intervals as sedentary time (< 53 counts), light‐intensity (LPA) (53–387 counts) or moderate‐ to vigorous‐intensity physical activity (MVPA) (≥ 388 counts) using published cut‐points [18]. Data from days 1 and 7 were excluded from analyses to ensure only data captured during the full 24‐h periods (defined as midnight to midnight) were considered. As in Ryan et al. [16], any days with > 10 h of total physical activity were deemed invalid and excluded from the analysis. For inclusion in the final analysis, participants had to provide at least three valid days of data (i.e., at least 16 h of wear time per day, with a minimum of 10 h of awake wear time) [19] including one weekend day. The average time spent in each behaviour was weighted at 5:2 for weekdays and weekend days, respectively [20, 21].

#### 2.2.3 Screen Time

Screen time was proxy‐reported by caregivers using a questionnaire. Caregivers were asked, for a usual weekday and for a usual weekend day, ‘How long on an average day does your child spend playing inactive video games?’, ‘How long on an average day does your child spend playing active video games?’ and ‘How many hours a day does your child spend watching television and/or videos/DVD?’. The range of potential responses was 0–7 h in 30‐min increments. Screentime hours were computed as the sum of the responses from the 3 questions, separately for weekdays and weekends. Weighted daily screentime data were calculated using the following formula ([weekday hours × 5] + [weekend hours × 2]/7 days). Prior to analysis, the weighted total screen time variable was truncated at a maximum of 18 h per day for the *n* = 17 participants who had > 18 h.

#### 2.2.4 Anthropometric Measurements

Measures included weight, height and waist circumference and the resultant calculations of body mass index (BMI, as weight in kilograms divided by height in meters squared) and BMI *z*‐score using Centers for Disease Control reference data [22, 23]. Waist‐to‐height ratio (expressed as a percent, i.e., waist (cm)/height (cm) × 100) was also calculated. Trained staff in all jurisdictions used standard instruments, including scales for weight (Seca 876), stadiometers for height (model PE‐AIM‐101; Perspective Enterprises) and tape measures for waist circumference (Seca 201). Before measuring children for the study, all measurers had to display good agreement compared with an expert [24], as determined per guidelines by Zerfas [25] during training sessions on anthropometry.

#### 2.2.5 Demographic and Other Participant Characteristics

Demographic information was reported by caregivers in a questionnaire, with assistance from a trained researcher. This included the child's age, sex and race or ethnicity according to the Office of Management and Budget categories [26]. Additional race and ethnicity subcategories were provided under Asian, Native Hawaiian and Pacific Islanders and American Indian/Alaska Native. An indigenous variable was developed from the Pacific Islander and Alaskan Native ethnicities, indicating the ethnicity indigenous to the jurisdiction in which the data were collected (Palau: Palauan, Yap: Yapese, Guam: Chamoru, Commonwealth of the Northern Mariana Islands: Chamorro and Carolinian, Chuuk: Chuukese, Pohnpei: Pohnpeian, Kosrae: Kosraean, Republic of Marshall Islands: Marshallese, American Samoa: Samoan, Hawai‘i: Native Hawaiian, Alaska: American Indian or Alaskan Native). For analyses, a binary Indigenous/Non‐indigenous variable was used. Education of caregiver was obtained in the following categories (never attended, attended grades 1–8, attended grades 9–11, high school diploma or General Educational Development, some college and 4‐year college) and was dichotomized into High School or less/ More than high school.

### 2.3 Statistical Analysis

All analyses were conducted in R software (version 4.3.2) using the compositions [27], zCompositions [28], factoextra [29], cluster [30] and lme4 [31] packages. Analyses were stratified by age (2–5 years and 6–8 years) to approximate pre‐school and elementary school age. Due to statistically significant sex interactions, all analyses were also stratified by sex. We conducted cluster analysis of multiple lifestyle variables comprising two compositions (macronutrient and movement behaviour) as well as total dietary energy intake and screen time. The macronutrient composition (% of total energy intake from saturated fat, unsaturated fat, protein, added sugar and other carbohydrates) was checked for zero values in any of the parts, before expressing them as a set of isometric log ratios, following compositional data analysis procedures. Seventy‐three (2%) participants had zero added sugar. These zeros were treated as rounded zeros with the assumption that if the participants were sampled for a long enough period or with a more sensitive measurement tool, they would eventually record some non‐zero value. The zeros were replaced by small imputed values using the expectation maximisation algorithm (lrEM) in the zCompositions package [28]. Similarly, the 24‐h movement behaviour composition (time spent in sleep, sedentary time, LPA and MVPA) was expressed as a set of isometric log ratios [32]. There were no zeros in any of the movement behaviours requiring replacement. As suggested by Haszard et al. [33], non‐wear time was assumed to occur during waking hours only and was filled in by apportioning the waking time sub‐composition (sedentary time, LPA and MVPA) so that, together with sleep, the complete composition summed to 1440 min.

Prior to cluster analysis, cluster input variables were scaled. Spherical scaling (to a multivariate variance of 1) was used for macronutrient composition and 24‐h movement behaviour composition. Spherical scaling is appropriate for compositional data as the solutions are invariant to permutations of the components [34]. Total energy intake and screen time were scaled by subtracting the arithmetic mean and dividing by the standard deviation of the age‐specific sample.

Due to the skewed nature of some of the cluster input variables, a partitioning around medoids (PAM) clustering algorithm was used [35]. PAM is more robust to outlying observations than K‐means algorithms. We used two metrics available in the Factoextra package [29] to determine the optimal number of clusters (Figures S2–S9). First, we plotted the within‐cluster sum of squares and visually identified where (at how many clusters) the ‘elbow’ was evident. We then used the silhouette plot to identify the largest average silhouette width. This encompasses two clustering criteria: separation (i.e., average distance to the closest other cluster) and compactness (i.e., average within‐cluster distance) [36]. When the metrics did not indicate the same optimal number of clusters (only for 6‐ to 8‐year‐old girls), we considered the meaningfulness of the cluster solutions to select which metric to use. Clusters were described by their sociodemographic characteristics and by their behavioural (diet, activity and time use) characteristics. The behavioural characteristics were expressed as *z*‐scores so that they were all on the same scale and presented relative to the grand mean of the sample, to facilitate visual comparisons between them. In an additional supplementary analysis, we observed how the Healthy Eating Index‐2020 [12] component scores for diet quality (derived from the dietary records) differed across the clusters.

Cluster membership (categorical variable) was used as a predictor in multilevel linear regression models, with BMI *z*‐score and waist‐to‐height ratio as the dependent variables. Random intercepts for communities, nested within jurisdiction, were used to account for the clustered sampling design. Models were adjusted for age, indigenous status and parental education. Model assumptions of homogeneity of variance, linearity and normality of residuals were determined to be satisfactory following visual inspection of diagnostic plots.

## 3 Results

A total of 1780 participants had complete and valid data and were included in the analyses (*n* = 1729 for waist‐to‐height ratio). The participant flow is presented in the Figure S1. Table 1 presents the characteristics of the included sample. For the overall sample, the compositional mean (calculated as the geometric means of each component, expressed as a percent of the total) of the macronutrient composition was: Saturated fat = 11%; Unsaturated fat = 21%; Protein = 15%; Added sugar = 2%; Other carbohydrates = 51% and for the movement behaviour, the compositional mean was: Sleep = 42%; Sedentary time = 26%; Light physical activity = 23% and MVPA = 9%. Included participants were of older age (5.55 vs. 5.29 years), had lower total energy intake (1747 vs. 1794 kCal/day), higher screen time (3.6 vs. 3.4 h), lower BMI *z*‐score (0.41 vs. 0.50) and lower waist‐to‐height ratio % (49.74 vs. 50.15). They also differed in their geographical spread across jurisdictions (see Table S1 for details).

**TABLE 1:** Participant characteristics.

Variable	All	2–5 years		6–8 years	
	*N* = 1780	*N* = 556	*N* = 567	*N* = 321	*N* = 336
Sex, *n* (%)		**Boys**	**Girls**	**Boys**	**Girls**
Boys	877 (49%)	556 (100%)	0 (0%)	321 (100%)	0 (0%)
Girls	903 (51%)	0 (0%)	567 (100%)	0 (0%)	336 (100%)
Age in years, mean (SD)	5.5 (1.6)	4.6 (1.0)	4.5 (1.0)	7.3 (0.8)	7.3 (0.8)
Indigenous status, *n* (%)					
No	471 (27%)	165 (30%)	146 (26%)	85 (26%)	75 (22%)
Yes	1309 (73%)	391 (70%)	421 (74%)	236 (74%)	261 (78%)
Parental education *n* (%)					
High school or less	1047 (59%)	323 (58%)	345 (61%)	189 (59%)	190 (57%)
More than high school	733 (41%)	233 (42%)	222 (39%)	132 (41%)	146 (43%)
Jurisdiction, *n* (%)					
Alaska	177 (10%)	72 (13%)	53 (9%)	24 (8%)	28 (8%)
American Samoa	246 (14%)	67 (12%)	76 (13%)	53 (17%)	50 (15%)
CNMI	311 (18%)	81 (15%)	94 (17%)	70 (22%)	66 (20%)
FAS	405 (23%)	151 (27%)	127 (22%)	59 (18%)	68 (20%)
Guam	318 (18%)	80 (14%)	97 (17%)	68 (21%)	73 (22%)
Hawai‘i	323 (18%)	105 (19%)	120 (21%)	47 (15%)	51 (15%)
Movement behaviours, mean (SD)					
Sleep (min/day)	590.9 (50.9)	597.2 (52.9)	591.7 (50.7)	580.7 (48.5)	588.7 (48.9)
Sedentary time (min/day)	369.4 (64.9)	359.8 (62.2)	362.1 (65.0)	385.9 (66.8)	382.0 (62.5)
Light physical activity (min/day)	325.8 (46.4)	317.4 (45.1)	337.0 (48.0)	315.8 (41.9)	330.4 (45.1)
MVPA^a^ (min/day)	125.8 (99.6; 153.2)	132.6 (104.8; 158.3)	114.2 (90.5; 137.6)	144.0 (117.2; 169.7)	121.6 (98.9; 145.2)
Screen time^a^ (h/day)	3.6 (2.0; 6.2)	3.5 (2.0; 6.1)	3.5 (1.9; 5.8)	4.5 (2.5; 7.0)	3.7 (2.1; 6.2)
Percent of dietary energy intake (*E*%), mean (SD)					
*E*% saturated fat	11.1 (2.3)	11.2 (2.4)	10.9 (2.2)	11.2 (2.4)	11.2 (2.2)
*E*% unsaturated fat	20.6 (3.7)	20.4 (3.7)	20.3 (3.7)	21.1 (3.5)	21.1 (3.6)
*E*% protein	15.4 (2.4)	15.2 (2.5)	15.4 (2.5)	15.5 (2.1)	15.7 (2.3)
*E*% added sugar	2.4 (1.0)	2.4 (1.1)	2.5 (1.0)	2.2 (1.0)	2.3 (1.0)
*E*% other carbohydrates	50.4 (5.8)	50.7 (5.9)	51.0 (6.0)	49.9 (5.4)	49.7 (5.8)
Total energy intake in kCal/day, Mean (SD)	1747 (483)	1696 (463)	1634 (452)	1932 (502)	1844 (478)
Anthropometric measures, mean (SD)					
BMI‐*z*	0.40 (1.09)	0.41 (1.13)	0.37 (1.01)	0.56 (1.12)	0.32 (1.11)
Waist‐to‐height ratio % *n* = 1730	49.7 (4.9)	50.4 (4.0)	50.8 (4.3)	48.6 (5.7)	48.0 (5.5)

*Note:* Summary statistics present as *n* (%) or mean (standard deviation), and median (interquartile range) if non‐normally distributed (denoted by ^a^). Some percentages do not add to 100% due to rounding.

Abbreviations: BMI‐*z*, body mass index *z*‐score; CNMI, Commonwealth of the Northern Mariana Islands; FAS, Freely Associated States; MVPA, moderate‐to vigorous‐intensity physical activity.

### 3.1 Cluster Characteristics

**FIGURE 1:**
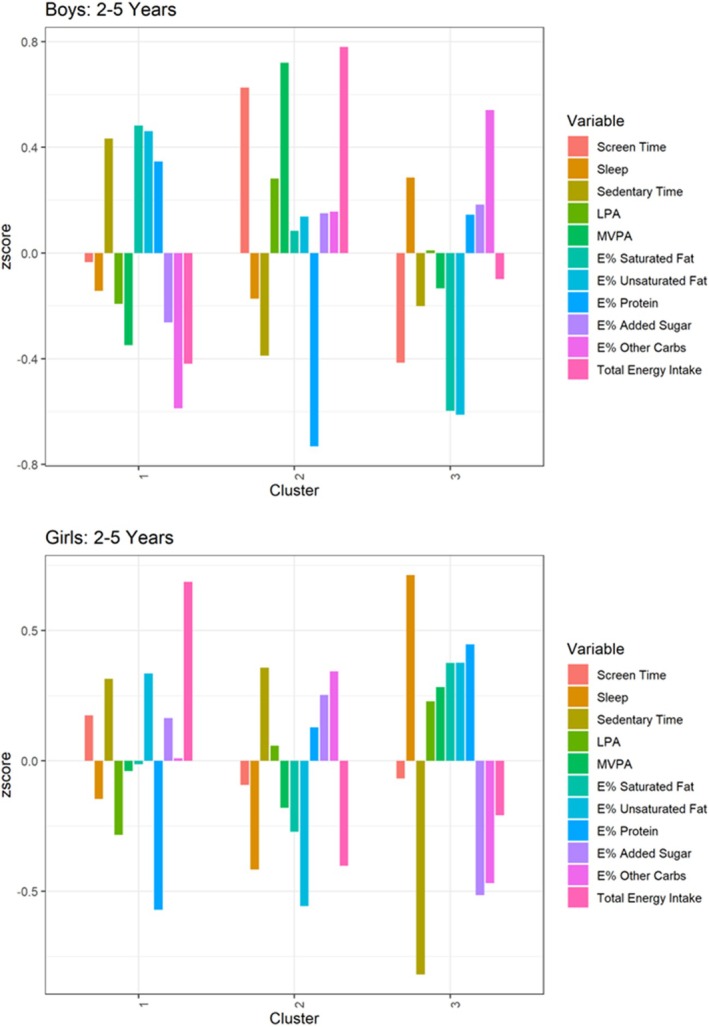
Cluster view for younger children (2–5 years): *Z*‐scores relative to the grand mean. Carbs, carbohydrates; *E*%, % of total energy intake; LPA, light physical activity; MVPA, moderate‐to‐vigorous physical activity.

**FIGURE 2:**
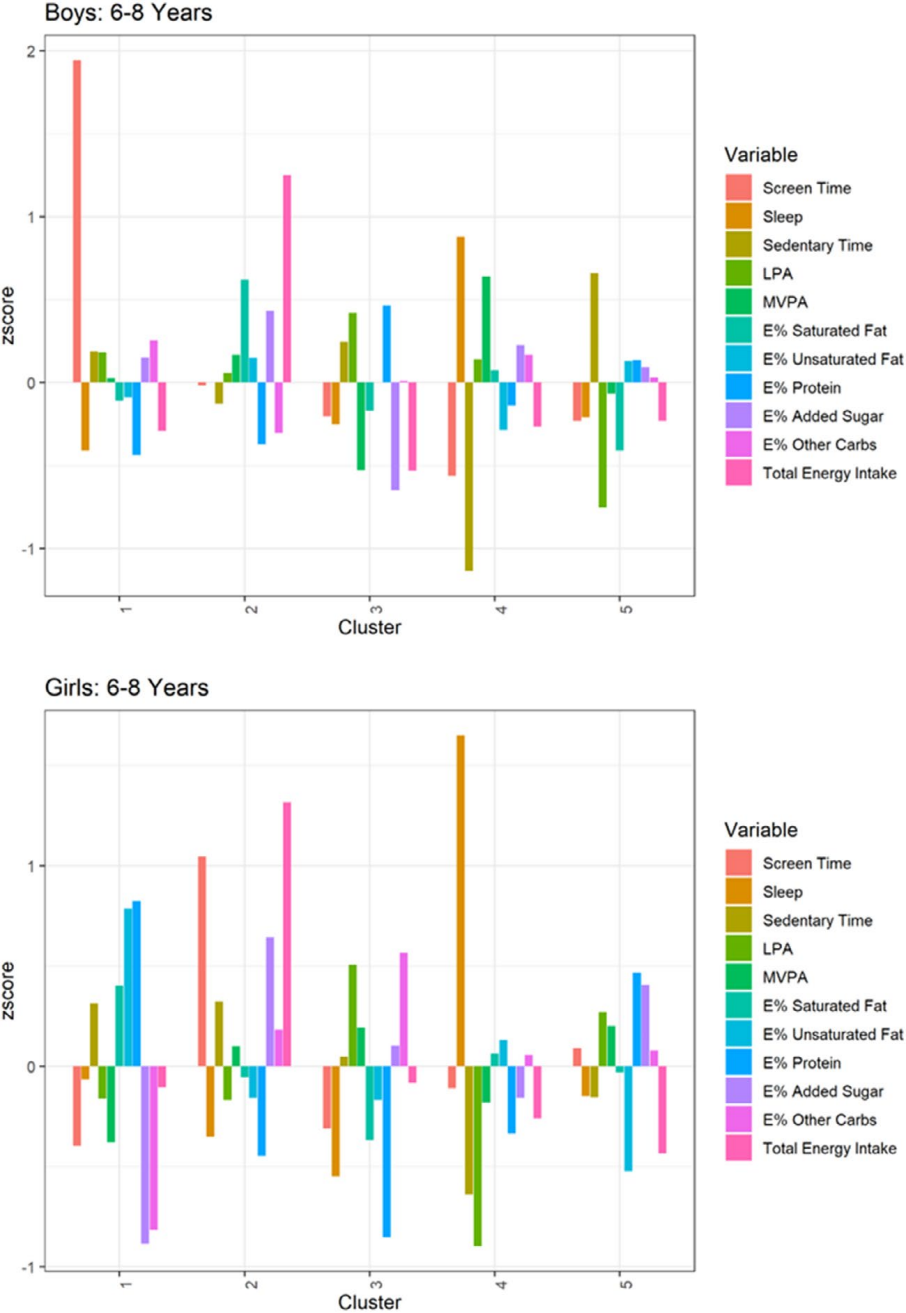
Cluster view for older children (6–8 years): *Z*‐scores relative to the grand mean. Carbs, carbohydrates; *E*%, % of total energy intake; LPA, light physical activity; MVPA, moderate‐to‐vigorous physical activity.

For both sexes, three clusters were identified in the younger age group (2–5 years) and five clusters in the older age group (6–8 years). The cluster labels are numbered and ordered to represent the anthropometrics of the group, so that a lower cluster number represents a higher average BMI *z*‐score i.e., Cluster 1 has the highest BMI *z*‐score according to the observed arithmetic means which are reported in Tables S1 and S2. Full descriptive statistics, including sociodemographic descriptives, of each of the clusters can also be found in Tables S2 and S3. Figures 1 and 2 present the average *z*‐scores of each of the lifestyle behaviour input variables across clusters, relative to the grand mean of the relevant subsample. The supplemental file contains additional figures (Figures S10–S12), with an alternative orientation (variable‐based perspective), and in a radial format.

#### 3.1.1 Ages 2–5 Years

Among boys, Cluster 1 was characterised by high sedentary time and low physical activity (light physical activity and MVPA) and a diet high in % of energy from fats and low in % of energy from carbohydrates and low total energy intake. Cluster 2 had high screen time, MVPA and energy intake and low % of energy from protein in their macronutrient composition. Cluster 3 had diets with highest % of energy from carbohydrates, longest sleep, lowest screen time and lowest % of energy from fat intake.

Among girls, Cluster 1 was characterised by a diet with high total energy intake and low % of energy from protein and relatively high screen time, high sedentary time and low light physical activity. Cluster 2 had low total energy intake and low % of energy from unsaturated fat, as well as short sleep and high sedentary time. Cluster 3 had long sleep, high physical activity (light and MVPA) and low sedentary time, as well as diets low in % of energy from carbohydrates (added sugar and other carbohydrates) and high in % of energy from protein and % of energy from fats (saturated and unsaturated).

#### 3.1.2 Ages 6–8 Years

Among boys, the most identifying feature of Cluster 1 was high screen time, along with short sleep and low % of energy from protein in their diet. Cluster 2 was characterised by high total energy intake with a high % of energy from saturated fats. Cluster 3 had diets with high % of energy protein and low % of energy from added sugar and low MVPA. Children in Cluster 4 had long sleep, as well as low sedentary time (including screen time) and high MVPA. Cluster 5 had high sedentary time, low light physical activity and relatively low % of energy from saturated fat intake.

Among girls, the most identifying features of Cluster 1 include macronutrient compositions with low % of energy from carbohydrates (added sugar and other carbohydrates) and high % of energy from protein and unsaturated fats. They also had low MVPA. Cluster 2 was characterised by high screen time and total energy intake, with high % of energy from added sugar and low % of energy from protein. Cluster 3 had diets with low % of energy from protein and relatively short sleep with high light physical activity. Children in Cluster 4 had long sleep, together with low sedentary time and light physical activity. Cluster 5 had macronutrient compositions low in % of energy from unsaturated fat, high in % of energy from protein and low in overall energy intake.

#### 3.1.3 Associations Between Lifestyle Clusters and Anthropometric Measures

**FIGURE 3:**
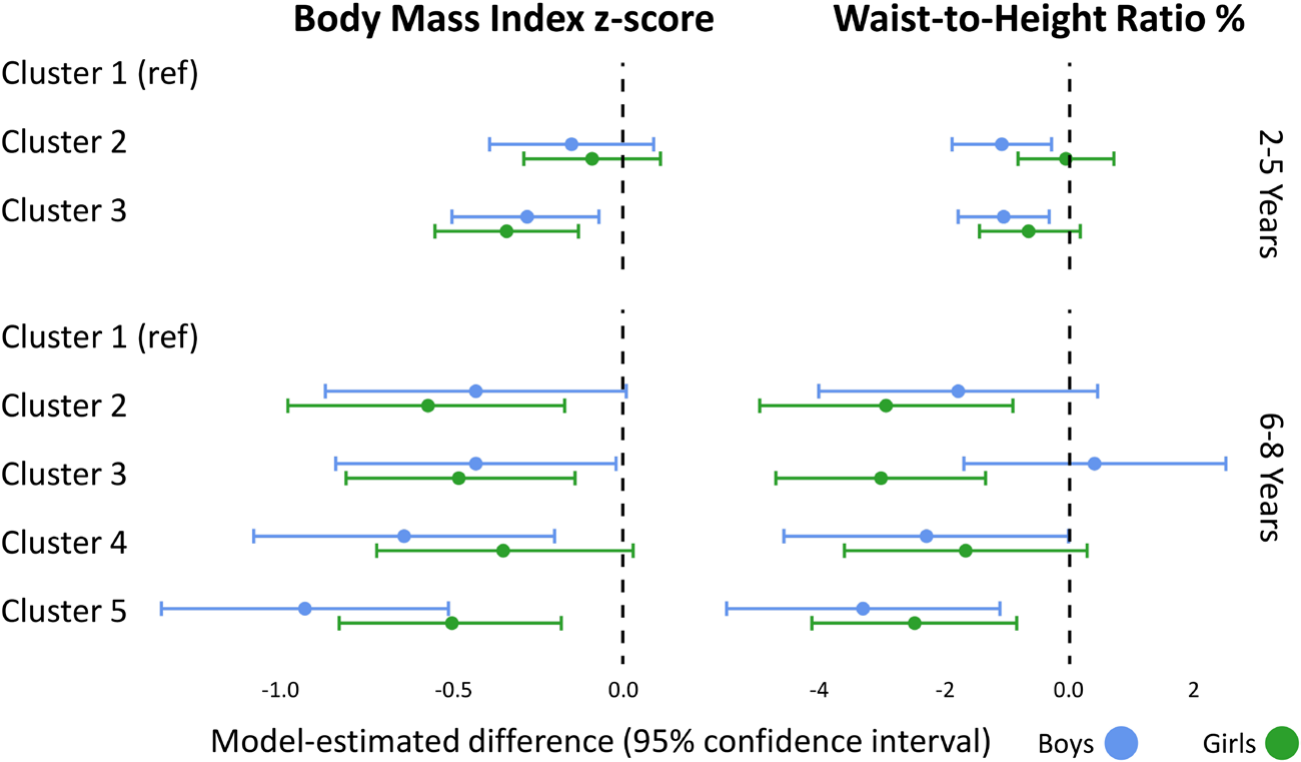
Relationship between cluster membership and anthropometric measures. All analyses were adjusted for child's age, indigenous status, parental education level and clustering at jurisdiction and community levels.

In multi‐level regression models for BMI *z*‐score, there were statistically significant differences by cluster membership for all sex and age groups (global *F* tests range: *p* < 0.001–0.04). For those aged 2–5 years, BMI z‐score was lower in Cluster 3 than in Cluster 1 (beta −0.28 [95% CI: −0.50, −0.07] and −0.34 [−0.55, −0.13], for boys and girls, respectively). For those aged 6–8 years, Cluster 1 (adjusted BMI *z*‐score = 1.08 [0.23] for boys and 0.64 [0.20] for girls) had a higher BMI *z*‐score than three of the four other clusters (*p* range < 0.001 to 0.05 for boys and *p* range 0.003 to 0.07 for girls). Similar patterns were observed for waist‐to‐height ratio, except associations were not statistically significant among girls aged 2–5 years (Figure 3, Table S4).

## 4 Discussion

We identified distinct lifestyle behaviour clusters among young children from Pacific Islander US‐affiliated jurisdictions. Membership in these lifestyle behaviour clusters was associated with differences in anthropometric measures.

The youngest children (aged 2–5 years) clustered into three lifestyle patterns which differed slightly between the sexes. Both boys and girls had a cluster characterised by high screen time, high total energy intake and low % of energy from protein (Cluster 2 in boys and Cluster 1 in girls). This lifestyle pattern also emerged among 6‐ 8‐year‐old girls (Cluster 2); but among 6‐ to 8‐year‐old boys, highest screen time and lowest % of energy from protein did not co‐occur with high energy intake (Cluster 1). The coexistence of high screen time and unhealthy diet (sometimes termed ‘Junk‐Food Screenies’) has been frequently observed in other studies applying cluster analyses to children's lifestyle behaviours [37], and this lifestyle pattern has previously been associated with poorer adiposity outcomes [8]. In our study, the cluster with highest screen time and lowest % of energy from protein intake also had the highest BMI *z*‐score in two subgroups (2–5 year old girls and 6‐ to 8‐year‐old boys) and the second highest BMI *z*‐score in the remaining subgroups (2‐ to 5‐year‐old boys and 6‐ to 8‐year‐old girls).

There are plausible mechanisms for screen‐based behaviours leading to increased adiposity as they are often accompanied by snacking behaviours [38], exposure to marketing for unhealthy foods [39] and lower energy expenditure [40]. These mechanisms are somewhat supported by our study, as increased energy intake usually co‐occurred with high screen time; however, the unremarkable contribution of % of energy from added sugar (except among 6‐ to 8‐year‐old girls) and % of energy from saturated fats to macronutrient composition in this cluster is unexpected. The relatively lower contribution % of energy from protein that is consistently observed to co‐occur with high screen time may indicate a trade‐off where the higher intake of the remaining macronutrient components leads to relatively lower % of energy from protein consumption and an increase in total energy intake [41].

The combination of high physical activity and low sedentary behaviour (and vice‐versa) has commonly been described in previous research, co‐occurring with mixed healthy and unhealthy diet behaviours [37]. Similar juxtaposed activity patterns were observed in our sample; higher physical activity (light and/or MVPA) coexisted with low sedentary time in 5 out of the 6 clusters observed among 2‐ to 5‐year‐olds and in 8 out of 10 clusters observed among 6‐ to 8‐year‐olds. Previous research has generally found that clusters with higher physical activity and lower sedentary time are associated with lower adiposity regardless of dietary intake [37]. For example, one study of *n* = 5701 children aged 9–11 years (slightly older than those in our sample) from 12 different countries (but not including countries/territories from the US‐Affiliated Pacific) found the lowest level of obesity among a cluster characterised by high physical activity and low sedentary time (‘Actives’) and the highest level of obesity among the cluster with high sedentary time and low physical activity (‘Sitters’) [8]. The same relationships were observed for some, but not all sex and age subgroups in our study.

Discrepancies with past research may be due to differences in operationalising dietary intake—in former studies, diet behaviour was assessed either by consumption of specific foods (e.g., sugar sweetened beverage consumption) or diet quality scores [37, 42]. We are not aware of any previous lifestyle cluster analysis that has considered the macronutrient composition as we have in our analysis. We performed an additional analysis to observe how diet quality for 13 food‐based components (operationalised as the Healthy Eating Index‐2020 component scores) differed across the clusters (see Tables S5 and S6). There were no clear patterns across the age and sex groups for how diet quality clustered together with other lifestyle behaviours, or how it was associated with obesity markers. It may be that the role of dietary intake in the relationship with adiposity is more pronounced and clearly observed when a more comprehensive measure of overall macronutrient composition and energy intake is used, as in our study.

Very few previous lifestyle clustering studies have included sleep duration, and those that did, have not reported clear patterns in how sleep co‐occurs with other behaviours [37, 42]. In our study, the clusters with the longest sleep duration consistently also had lowest sedentary time (except among 2‐ to 5‐year‐old boys, where it was second lowest). Dietary patterns co‐occurring with long sleep/low sedentary time are less clear. Among 2‐ to 5‐year‐olds, boys with long sleep/low sedentary time also had lowest % of energy from fats (saturated and unsaturated) and girls also had the lowest % of energy from added sugar and other carbohydrates. These combinations appear to reflect a better macronutrient composition. However, the same was not observed among 6‐ to 8‐year‐olds, with no clear dietary patterns co‐occurring with long sleep/low sedentary time. The clusters with longest sleep/low sedentary time had the lowest BMI *z*‐score in 2‐ to 5‐year‐olds and second lowest among 5‐ to 8‐year‐olds, in line with systematic review evidence of the protective role of sufficient sleep for childhood obesity [43].

### 4.1 Strengths and Weaknesses

This is the first study to describe diet and movement behaviour clusters among children from Pacific Islander US‐affiliated jurisdictions, who represent an understudied population among those with the highest risk of obesity in the world. Strengths of the study include the comprehensive diet assessment, derived from a validated dietary record methodology, as well as device measured 24‐h movement behaviours. The use of novel compositional data analytics enabled the inherent relative nature of the macronutrient and movement behaviour compositions to be incorporated in the clustering algorithm.

Limitations to be considered when interpreting the findings include the cross‐sectional nature of the data, meaning causality between cluster membership and obesity measures cannot be ascertained. Genetically informed studies may assist in elucidating causality in future research. Diet and activity information were gathered across the same time period, meaning they could have potentially interfered with each other; however, this is unlikely to have introduced systematic bias as the 2 days for diet measurement were randomly selected. Many participants (*n* = 3721) were excluded due to missing data, and included participants differed from excluded participants in a number of characteristics (age, jurisdiction, total energy intake, screen time and BMI *z*‐score), meaning potential selection bias could limit generalizability. The screen time question was reported by parents in crude increments (30 min), and possible responses could range from 0 to 7 h summed across three items, thus this variable could have a maximum of 21 h/day, which appears infeasible as a habitual behaviour on an ongoing basis. We truncated the screen time variable at a maximum of 18 h, as this would still allow for 6 h sleep. As this variable was not mutually exclusive of the other movement behaviour variables, it could be plausible that some children use screens concurrently with other daily activities, or in cultural settings where the television is on in the room where children sleep and where there are small family sleeping spaces. Even 18 h of screen time per day appears unlikely to be sustainable, nonetheless, responses of such a high magnitude may be an important marker for extremely high screen use, such as in the aforementioned cultural setting or video game addiction.

### 4.2 Implications

While cluster membership was a predictor of BMI *z*‐score in all age and sex subgroups, no one pattern of lifestyle behaviours was consistently associated with the highest or lowest anthropometry measures across the subgroups. This implies that a range of lifestyle choices across diet, activity and sleep may be able to achieve improvement in adiposity. If the equivalence of behaviour change options are confirmed by future longitudinal studies, children and families may be able to choose which behaviour changes are best suited to their preferences and contexts. In the future, interventions may provide a ‘menu’ of lifestyle behaviour changes which could lead to equivalent adiposity outcomes. If the effect sizes observed in our study reflect causal relationships, shifting lifestyles towards a healthier combination of behaviours could have a substantial impact on child obesity. The average zBMI of clusters with the highest obesity measures were around a half a standard deviation higher than the average zBMI of the remaining clusters, with almost one standard deviation (0.93) separating the highest and lowest zBMI clusters among boys aged 6–8 years.

## 5 Conclusion

This study highlights the importance of dietary intake, sleep and physical activity for young children in the US‐affiliated Pacific and adds support for the unfavourable associations of excessive screen time, particularly when combined with high total energy intake and/or short sleep and low MVPA. We found that a variety of lifestyle patterns are associated with healthier adiposity outcomes, indicating there may be flexibility in which lifestyle behaviours could be targeted in intervention programs.

## Author Contributions

**Dorothea Dumuid:** conceptualization, investigation, writing – original draft, methodology, visualization, writing – review and editing, formal analysis. **Ashley B. Yamanaka:** conceptualization, investigation, funding acquisition, writing – review and editing, project administration, resources, data curation. **Kar Hau Chong:** conceptualization, investigation, methodology, writing – review and editing. **Anthony D. Okely:** conceptualization, investigation, methodology, writing – review and editing. **Lynne R. Wilkens:** conceptualization, investigation, funding acquisition, methodology, writing – review and editing. **Yurii B. Shvetsov:** conceptualization, investigation, methodology, writing – review and editing. **Chloe P. Lozano:** conceptualization, investigation, methodology, writing – review and editing. **Rachel Novotny:** conceptualization, investigation, funding acquisition, methodology, writing – review and editing, project administration, data curation, supervision.

## Conflicts of Interest

The authors declare no conflicts of interest.

## Supporting information


Appendix S1
