# Supplementary material for: Diet, Activity and Sleep Clusters Associated With Obesity Markers of Children in the US‐Affiliated Pacific
Source: Acta Paediatr. 2025 Feb 24;114(7):1642–52. doi: 10.1111/apa.70012 (PMC12147416; doi:10.1111/apa.70012)
Supplement: Supplementary file 1 — Appendix S1 [file APA-114-1642-s001.pdf]

Supplementary Material for:

Diet, activity and sleep clusters associated with obesity markers of children in the US-Affiliated Pacific

Authors: Dorothea Dumuid[a], Ashley B. Yamanaka[b], Kar Hau Chong[c], Anthony D. Okely[c], Lynne R. Wilkens[e], Yurii B. Shvetsov[e], Chloe P. Lozano[e], Rachel Novotny[b]

[a]Alliance for Research in Exercise, Nutrition and Activity, Allied Health & Human Performance, University of South Australia, Adelaide, Australia. Email: dot.dumuid@unisa.edu.au

[b]College of Tropical Agriculture and Human Resources, University of Hawaii at Manoa, Honolulu, Hawaii, 96822, USA

[c]School of Health and Society, Early Start, Faculty of the Arts, Social Sciences and Humanities, University of Wollongong, New South Wales, Australia

[e]Cancer Center, University of Hawaii at Manoa, Honolulu, Hawaii, 96813, USA

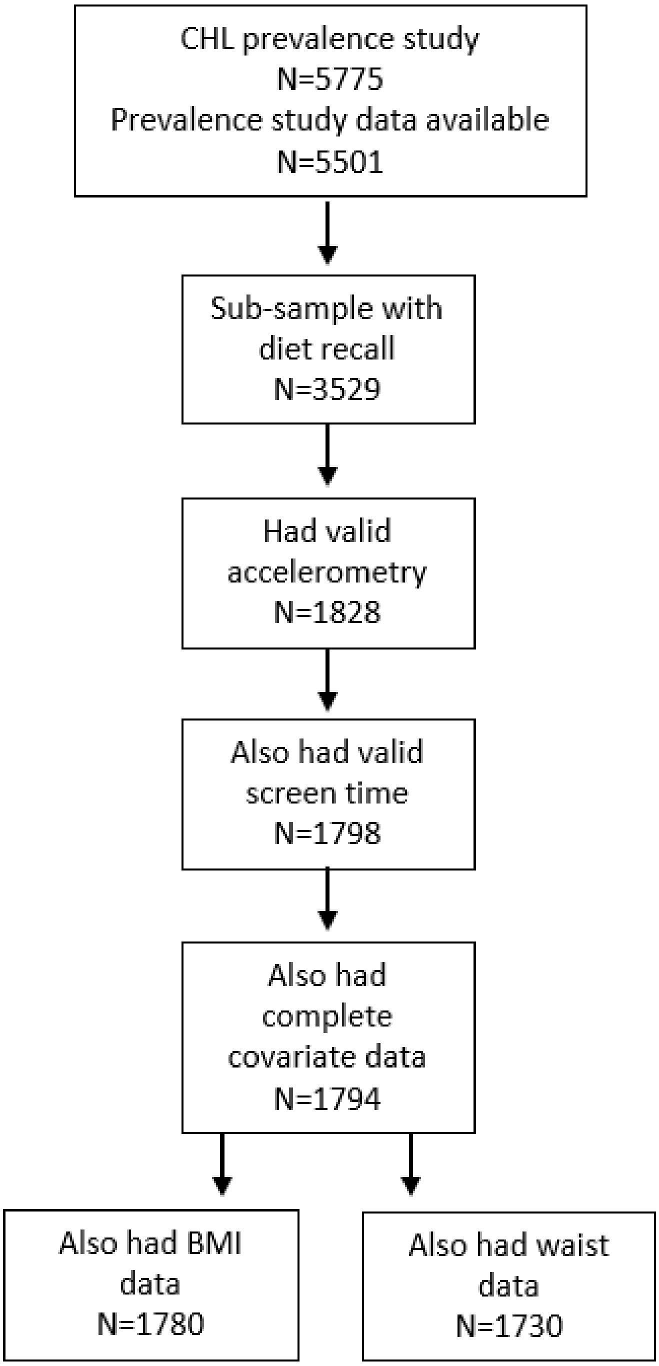

Figure S1. Participant flow

BMI = body mass index

Determining the appropriate number of clusters (elbow plots and silhouette plots).

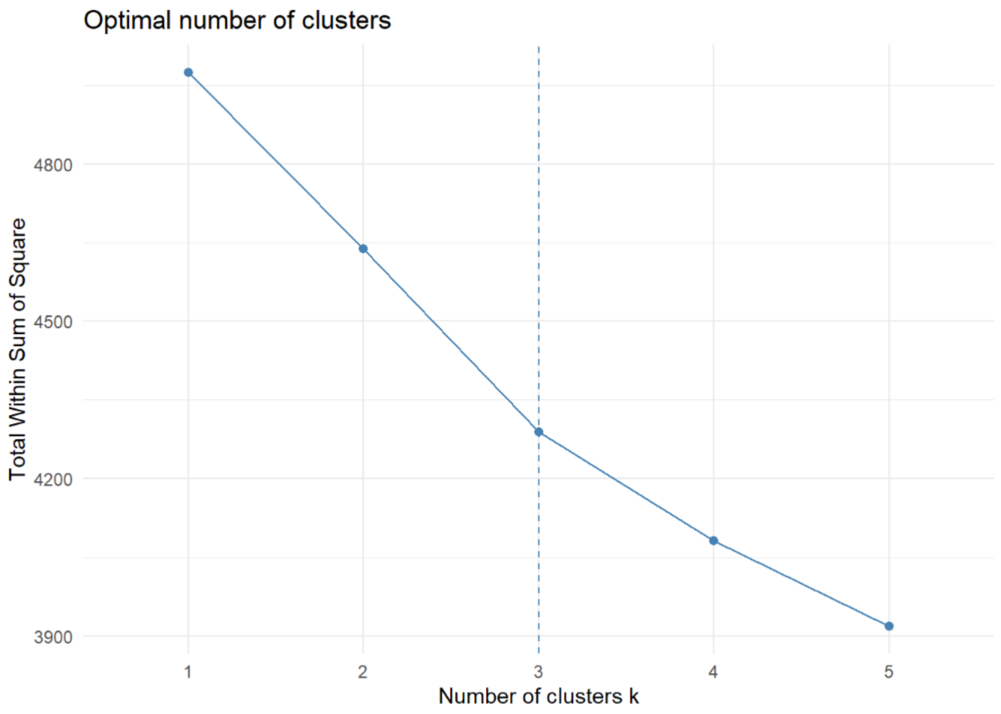

Figure S2. Elbow plot for 2–5-Year-Old Boys

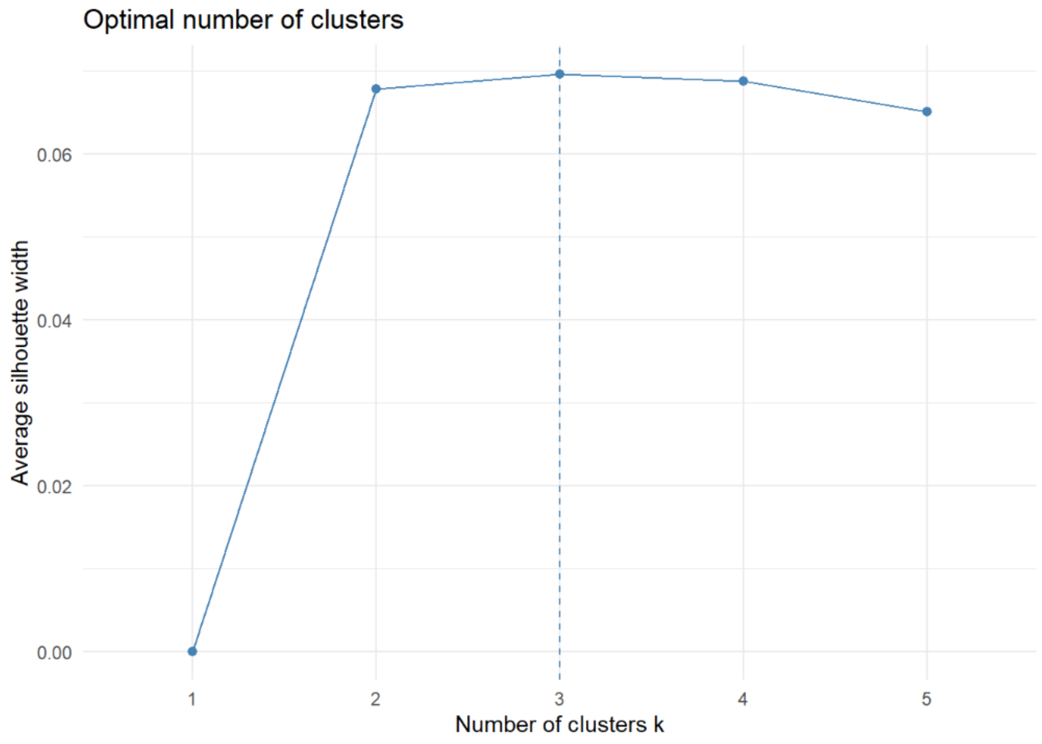

Figure S3. Silhouette plot for 2–5-Year-Old Boys

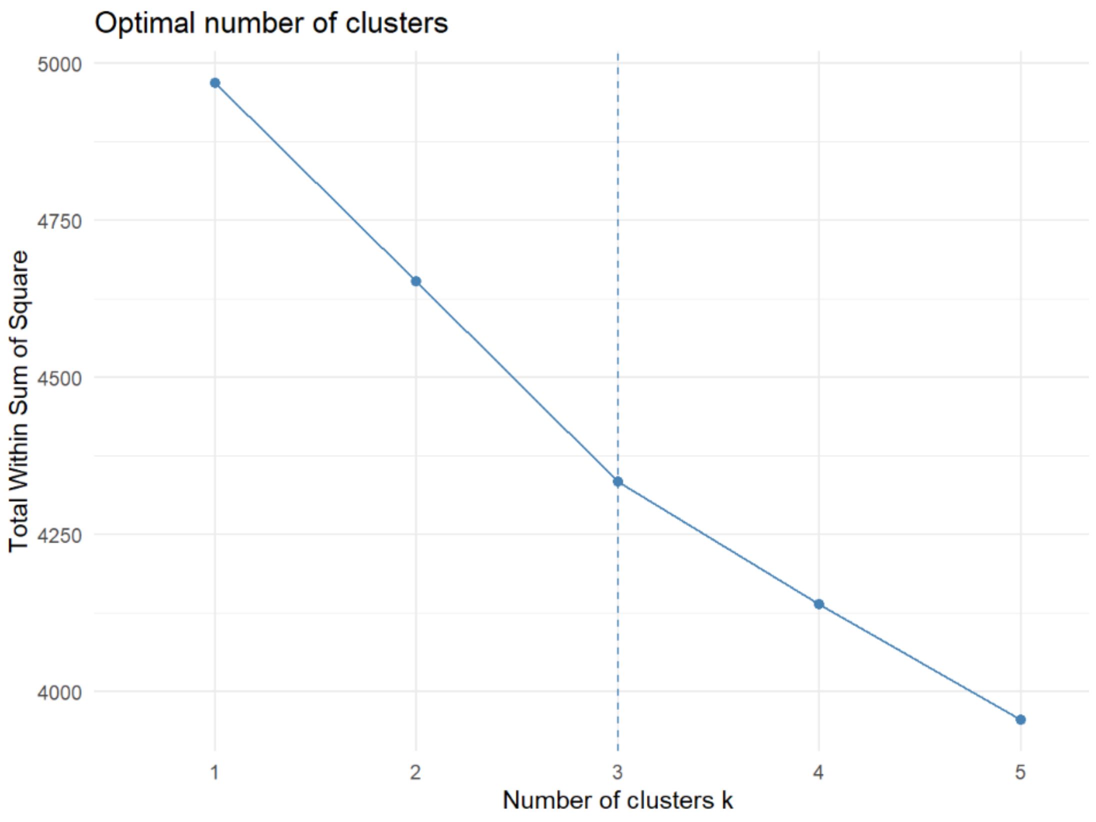

Figure S4. Elbow plot for 2–5-Year-Old Girls

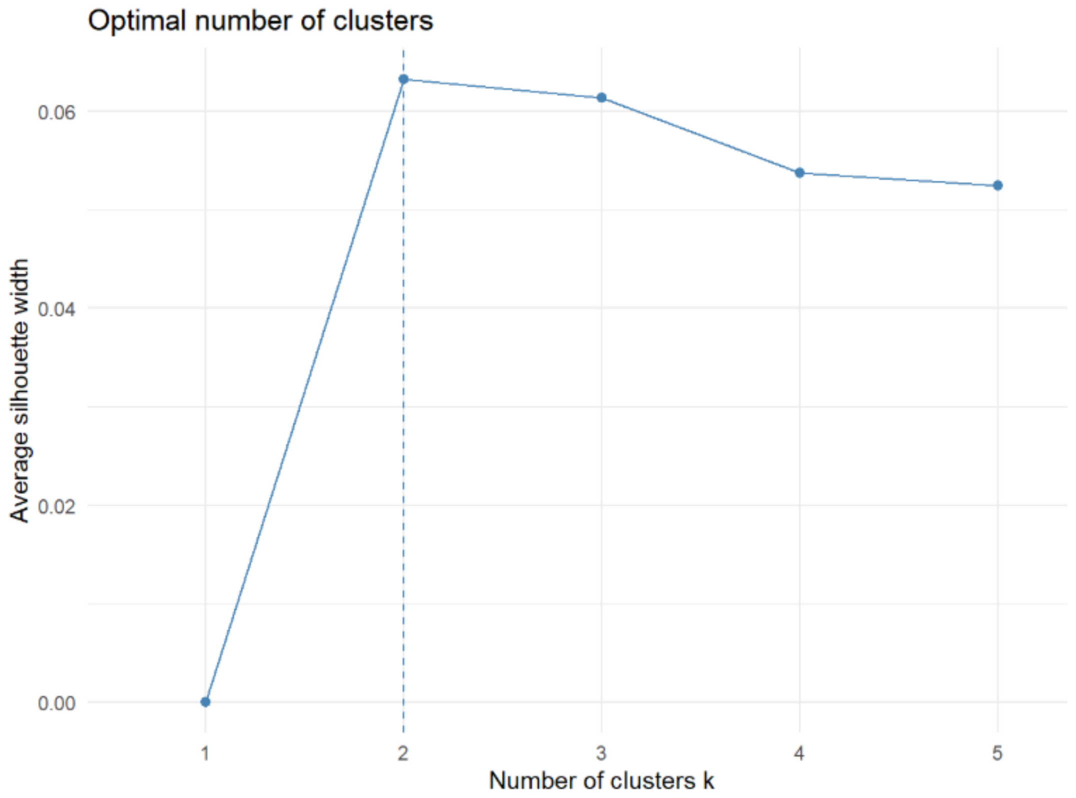

Figure S5. Silhouette plot for 2–5-Year-Old Girls

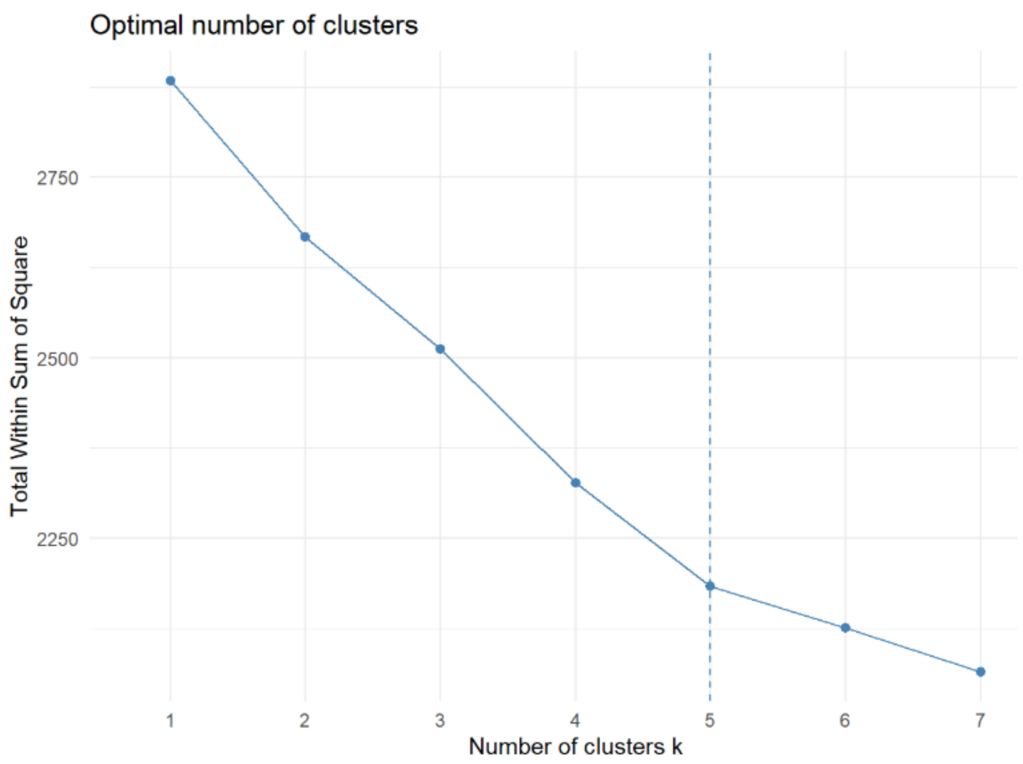

Figure S6. Elbow plot for 6–8-Year-Old Boys

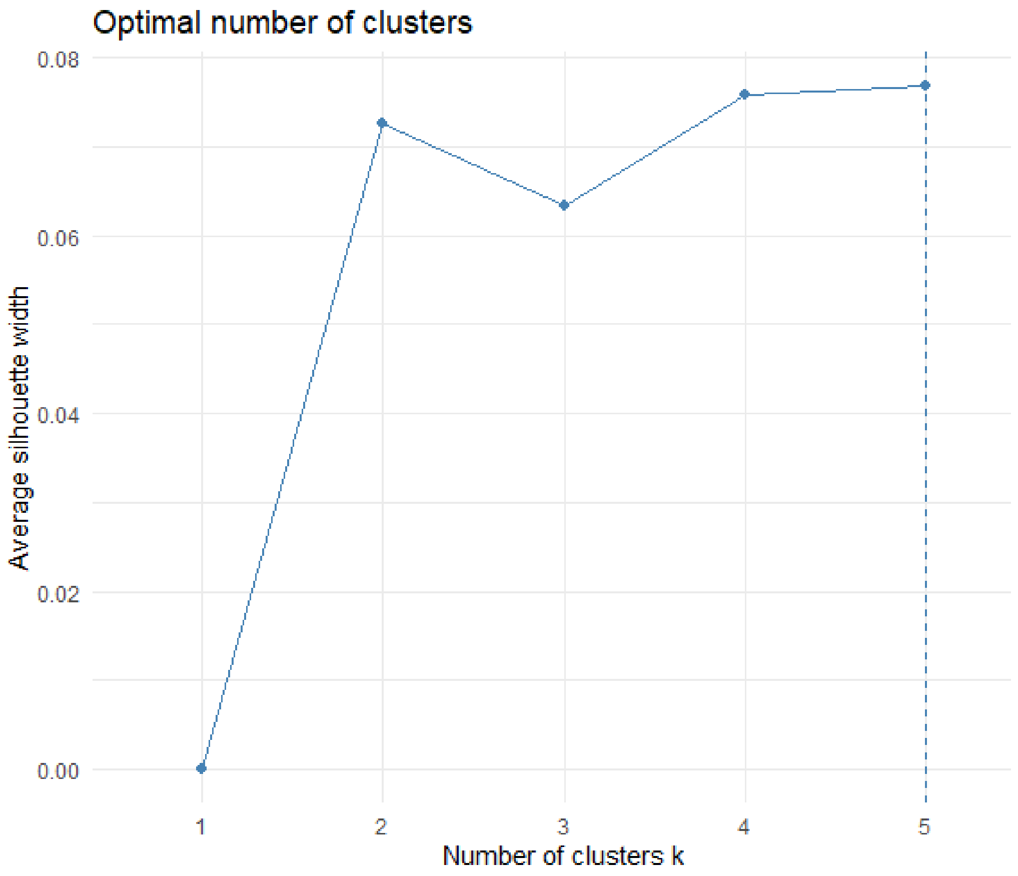

Figure S7. Silhouette plot for 6–8-Year-Old Boys

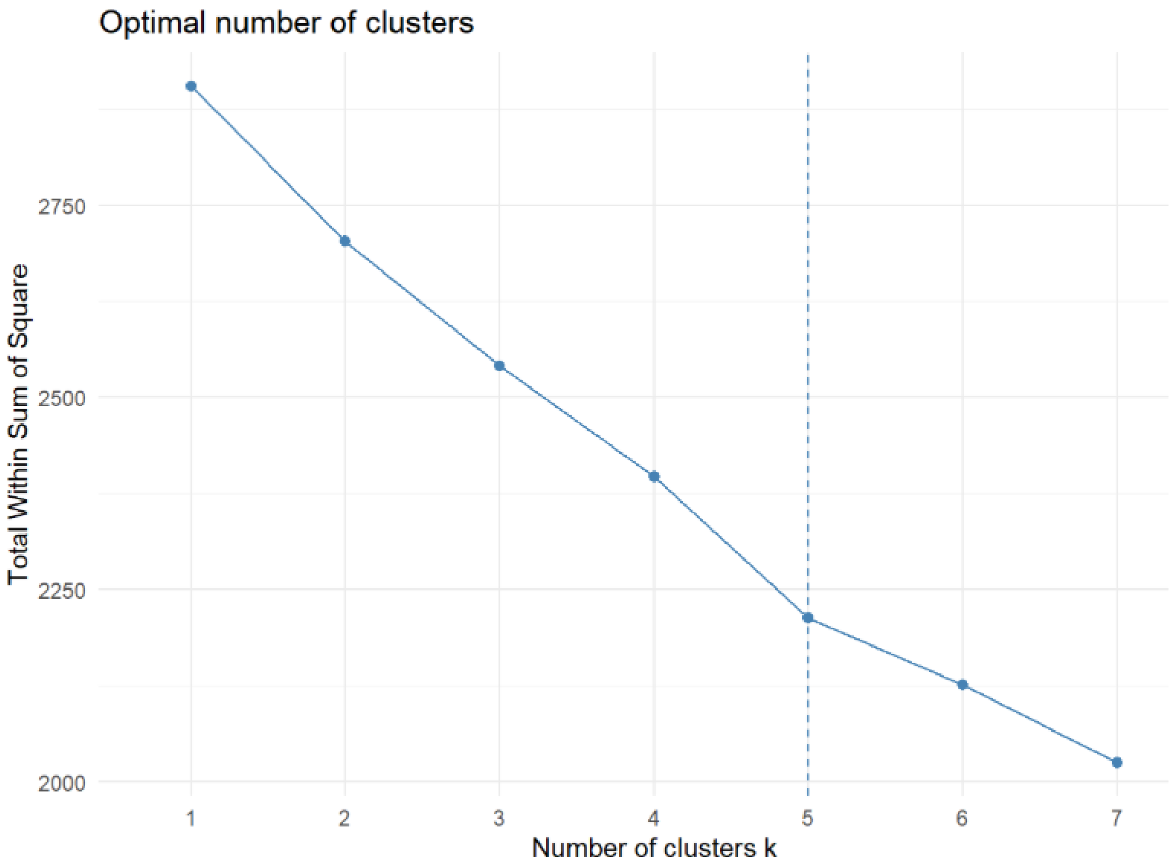

Figure S8. Elbow plot for 6–8-Year-Old Girls

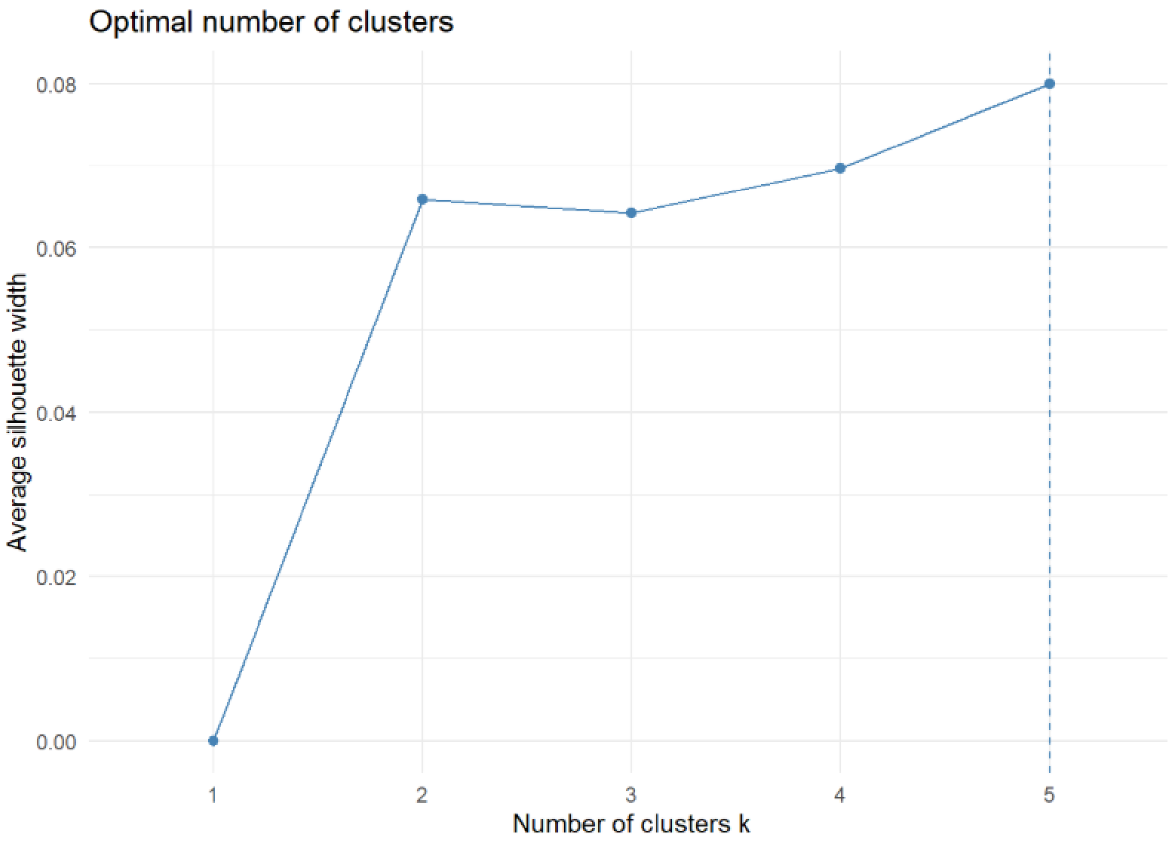

Figure S9. Silhouette plot for 6–8-Year-Old Girls

Table S1. Differences between included and excluded participants.

	Included	Excluded	*p*
	N=1780	N=3721	
Sex = Female, n (%)	903 (50.7)	1783 (47.9)	0.054
Age in years, mean (SD)	5.55 (1.63)	5.29 (1.64)	<0.001
Indigenous status = Yes (%)	1309 (73.5)	2689 (72.3)	0.337
Parental education = More than school (%)	733 (41.2)	1433 (38.6) $^{n=3708}$	0.077
Jurisdiction, n (%)			P<0.001
Alaska	177 (9.9)	488 (13.1)	
American Samoa	246 (13.8)	724 (19.5)	
CNMI	311 (17.5)	592 (15.9)	
FAS	405 (22.8)	765 (20.6)	
Guam	318 (17.9)	537 (14.4)	
Hawaii	323 (18.1)	615 (16.5)	
Percent of energy (E%) intake, mean (SD)			
E% Saturated fat	11.12 (2.32)	11.02 (2.39) $^{n=1751}$	0.190
E% Unsaturated fat	20.62 (3.65)	20.45 (4.02) $^{n=1751}$	0.179
E% Protein	15.42 (2.39)	15.45 (2.66) $^{n=1751}$	0.749
E% Added sugar	2.38 (1.03)	2.32 (1.10) $^{n=1751}$	0.111
E% Other carbohydrates	50.45 (5.82)	50.76 (6.35) $^{n=1751}$	0.134
Total energy intake (kCal/day), mean (SD)	1747.26 (483.27)	1794.68 (513.26) $^{n=1751}$	0.005
Movement behaviors, mean (SD)			
Sleep (min/d)	590.9 (50.94)	586.9 (47.4) $^{n=267}$	0.671
Sedentary time (min/d)	369.43 (64.92)	373.34 (66.97) $^{n=267}$	0.661
Light physical activity (min/d)	325.78 (46.37)	321.39 (48.28) $^{n=267}$	0.081
MVPA, (min/d)	125.8 (99.6; 153.2)	124.0 (97.5; 157.8) $^{n=267}$	0.988
Screen time (h/d)	3.6 (2.0; 6.2)	3.4 (1.7; 6.0) $^{n=2535}$	0.002
BMI-z	0.41 (1.09)	0.50 (1.11) $^{n=2450}$	0.004
Waist-to-height ratio %	49.74 (4.88) $^{n=1730}$	50.15 (5.00) $^{n=3571}$	0.003

Abbreviations: CNMI=Commonwealth of the Northern Mariana Islands; FAS=Freely Associated States; MVPA=moderate-to vigorous-intensity physical activity; BMI-z=body mass index z-score. NB p value is for test of differences between included and excluded participants, via t-tests for continuous normally distributed variables, Mann-Whitney U test for continuous non-normally distributed variables (MVPA and screen time), and Chi square for categorical variables.

Table S2. Characteristics of clusters: ages 2-5 years

Variable	Boys 2-5 y<br>**Cluster 1**, N = 218	 **Cluster 2**, N = 142	 **Cluster 3**, N = 196	Girls 2-5 y<br>**Cluster 1**, N = 180	 **Cluster 2**, N = 221	 **Cluster 3**, N = 166
**BMI z-score**	0.50 (1.17)	0.40 (1.17)	0.32 (1.05)	0.55 (0.96)	0.40 (1.02)	0.14 (1.02)
**Waist-to-Height (%)**	51.2 (4.5)	49.7 (4.0)	50.2 (3.3)	50.7 (4.2)	51.0 (4.4)	50.5 (4.3)
**Overweight/Obese**						
No	155 (71%)	105 (74%)	147 (75%)	122 (68%)	169 (76%)	134 (81%)
Yes	63 (29%)	37 (26%)	49 (25%)	58 (32%)	52 (24%)	32 (19%)
**Screen Time[a] (min/d)**	3.8 (3.8)	6.2 (7.1)	2.5 (2.9)	4.0 (4.5)	3.1 (3.5)	3.3 (3.8)
**Movement behavior composition (arithmetic means)**						
**Sleep (min/d)**	589.6 (51.6)	588.0 (48.8)	612.2 (54.0)	584.3 (47.4)	570.6 (40.6)	627.9 (47.2)
**Sedentary Time (min/d)**	386.8 (59.0)	335.7 (55.4)	347.3 (59.4)	382.6 (55.3)	385.4 (60.5)	308.9 (47.5)
**Light Physical Activity (min/d)**	308.7 (43.5)	330.1 (37.2)	317.8 (49.9)	323.4 (47.7)	339.8 (51.3)	347.9 (40.0)
**MVPA[a] (min/d)**	118.8 (48.2)	156.6 (46.6)	126.7 (63.5)	113.4 (47.5)	105.0 (45.0)	122.2 (50.3)
**Movement behavior composition (compositional means)**						
**Sleep (%)**	42.3	41.7	44.0	42.0	41.0	44.8
**Sedentary Time (%)**	27.6	23.6	24.7	27.3	27.5	21.8
**Light Physical Activity (%)**	22.0	23.3	22.6	23.0	24.2	24.7
**MVPA (%)**	8.1	11.3	8.7	7.7	7.3	8.6
**Macronutrient composition (arithmetic means)**						
**E% Saturated Fat**	12.4 (2.2)	11.4 (2.3)	9.8 (1.9)	10.9 (2.1)	10.3 (2.2)	11.7 (2.3)
**E% Unsaturated Fat**	22.1 (3.4)	20.9 (3.5)	18.1 (3.0)	21.5 (3.4)	18.3 (2.9)	21.7 (3.6)
**E% Protein**	16.1 (2.2)	13.5 (1.7)	15.6 (2.6)	13.9 (2.0)	15.7 (2.3)	16.5 (2.6)
**E% Added Sugar**	2.1 (0.9)	2.6 (1.2)	2.6 (1.1)	2.6 (0.9)	2.7 (1.0)	1.9 (1.0)
**E% Other Carbohydrates**	47.2 (5.0)	51.6 (5.0)	53.9 (5.3)	51.0 (5.3)	53.0 (5.5)	48.2 (6.1)
**Macronutrient composition (geometric means)**						
**E% Saturated Fat**	12.4	11.4	9.7	10.8	10.2	11.7
**E% Unsaturated Fat**	22.1	20.8	18.1	21.5	18.2	21.7
**E% Protein**	16.1	13.5	15.6	14.0	15.7	16.6
**E% Added Sugar**	1.9	2.3	2.3	2.5	2.5	1.6
**E% Other Carbohydrates**	47.5	52.0	54.3	51.3	53.4	48.5
**Total Energy intake (kCal)**	1,501 (342)	2,058 (523)	1,650 (382)	1,946 (442)	1,453 (347)	1,541 (414)
**Demographics**						
**Age (y)**	4.70 (3.78, 5.32)	4.96 (4.29, 5.55)	4.70 (3.78, 5.39)	4.88 (3.77, 5.42)	4.72 (3.85, 5.35)	4.71 (3.66, 5.25)
**Indigenous**						
No	74 (34%)	35 (25%)	56 (29%)	45 (25%)	50 (23%)	51 (31%)
Yes	144 (66%)	107 (75%)	140 (71%)	135 (75%)	171 (77%)	115 (69%)
**Caregiver education level**						
High school or less	132 (61%)	91 (64%)	100 (51%)	116 (64%)	130 (59%)	99 (60%)
More than school	86 (39%)	51 (36%)	96 (49%)	64 (36%)	91 (41%)	67 (40%)
**Jurisdiction**						
Alaska	33 (15%)	2 (1.4%)	37 (19%)	14 (7.8%)	20 (9.0%)	19 (11%)
American Samoa	18 (8.3%)	24 (17%)	25 (13%)	45 (25%)	16 (7.2%)	15 (9.0%)

CNMI	35 (16%)	24 (17%)	22 (11%)	29 (16%)	25 (11%)	40 (24%)
FAS	53 (24%)	40 (28%)	58 (30%)	23 (13%)	62 (28%)	42 (25%)
Guam	32 (15%)	29 (20%)	19 (9.7%)	39 (22%)	36 (16%)	22 (13%)
Hawaii	47 (22%)	23 (16%)	35 (18%)	30 (17%)	62 (28%)	28 (17%)

All values are n (%) or mean (SD), or median (IRQ) if not normally distributed, indicated by [a]
Abbreviations: BMIz = body mass index z-score, MVPA = moderate-to-vigorous physical activity, CNMI = Commonwealth of the Northern Mariana Islands; FAS = Freely Associated States; E% = % of daily energy intake

Table S3. Characteristics of clusters: ages 6-8 years

Variable	Boys 6-8 y Cluster 1, N = 35	Cluster 2, N = 70	Cluster 3, N = 85	Cluster 4, N = 58	Cluster 5, N = 72	Girls 6-8 y Cluster 1, N = 76	Cluster 2, N = 49	Cluster 3, N = 77	Cluster 4, N = 47	Cluster 5, N = 87
**BMIz**	1.02 (0.88)	0.70 (1.00)	0.59 (1.23)	0.36 (1.16)	0.32 (1.10)	0.57 (1.15)	0.31 (1.14)	0.29 (1.06)	0.24 (1.06)	0.17 (1.10)
**Waist-to-height (%)**	50.0 (5.9)	48.3 (6.1)	50.0 (6.6)	47.5 (3.8)	47.2 (4.7)	49.6 (5.7)	47.0 (5.8)	47.2 (4.8)	48.1 (5.7)	47.8 (5.6)
**Overweight/Obese**										
No	20 (57%)	48 (69%)	58 (68%)	43 (74%)	54 (75%)	49 (64%)	33 (67%)	60 (78%)	39 (83%)	71 (82%)
Yes	15 (43%)	22 (31%)	27 (32%)	15 (26%)	18 (25%)	27 (36%)	16 (33%)	17 (22%)	8 (17%)	16 (18%)
**Screen Time[a] (min/d)**	13.0 (3.5)	4.9 (4.4)	4.6 (3.4)	2.9 (2.4)	3.6 (3.9)	3.0 (2.8)	8.1 (4.5)	3.0 (2.6)	3.3 (4.0)	4.2 (4.1)
**Movement behavior composition (arithmetic means)**										
**Sleep (min/d)**	561.0 (36.5)	580.9 (45.6)	568.6 (42.6)	623.4 (47.5)	570.7 (44.4)	585.5 (32.6)	571.5 (37.0)	561.8 (34.0)	669.3 (42.3)	581.5 (34.6)
**Sedentary Time (min/d)**	398.8 (55.5)	377.4 (57.6)	402.5 (56.8)	310.2 (40.3)	430.2 (55.7)	401.7 (51.5)	402.3 (56.5)	385.0 (52.9)	342.0 (81.3)	372.3 (59.9)
**Light Physical Activity (min/d)**	323.3 (33.6)	318.1 (41.6)	333.3 (39.0)	321.6 (39.2)	284.1 (34.5)	323.2 (40.1)	322.8 (39.6)	353.2 (40.4)	290.0 (50.9)	342.6 (34.9)
**MVPA[a] (min/d)**	142.4 (36.1)	150.4 (59.9)	122.6 (46.5)	160.0 (51.1)	143.8 (45.8)	106.8 (36.6)	123.8 (30.3)	126.6 (44.4)	106.1 (61.8)	128.8 (36.2)
**Movement behavior composition (compositional means)**										
**Sleep (%)**	39.5	41.0	40.1	44.0	40.2	41.5	40.4	39.5	48.2	41.0
**Sedentary Time (%)**	27.9	26.4	28.2	21.8	30.2	28.3	28.2	26.9	24.1	26.0
**Light Physical Activity (%)**	22.7	22.3	23.4	22.6	19.9	22.8	22.7	24.7	20.5	24.1
**MVPA (%)**	10.0	10.3	8.3	11.7	9.6	7.4	8.7	8.8	7.2	8.9
**Macronutrient composition (arithmetic means)**										
**E% Saturated Fat**	10.9 (2.1)	12.7 (2.7)	10.8 (2.2)	11.4 (2.3)	10.2 (1.8)	12.1 (2.1)	11.1 (1.7)	10.4 (2.0)	11.4 (2.0)	11.2 (2.4)
**E% Unsaturated Fat**	20.8 (3.4)	21.7 (3.1)	21.1 (4.1)	20.1 (3.4)	21.6 (3.1)	23.9 (2.9)	20.5 (2.5)	20.5 (3.2)	21.5 (3.6)	19.2 (3.5)
**E% Protein**	14.6 (1.9)	14.7 (1.8)	16.5 (2.1)	15.2 (1.8)	15.8 (2.4)	17.6 (2.0)	14.7 (1.6)	13.7 (1.3)	14.9 (1.7)	16.8 (2.0)
**E% Added Sugar**	2.4 (0.9)	2.7 (0.9)	1.6 (0.9)	2.5 (0.8)	2.3 (0.9)	1.4 (0.6)	3.0 (1.0)	2.4 (0.9)	2.2 (0.9)	2.7 (0.9)
**E% Other Carbohydrates**	51.3 (4.4)	48.2 (4.8)	49.9 (6.2)	50.8 (5.8)	50.1 (4.7)	45.0 (4.8)	50.8 (4.0)	53.0 (4.8)	50.0 (5.0)	50.2 (6.0)
**Macronutrient composition (compositional means)**										

**E% Saturated Fat**	10.9	12.6	10.7	11.3	10.1	12.0	11.0	10.3	11.3	11.0
**E% Unsaturated Fat**	20.8	21.7	21.0	20.1	21.6	23.9	20.5	20.4	21.4	19.1
**E% Protein**	14.6	14.8	16.7	15.3	15.8	17.7	14.7	13.8	15.0	16.9
**E% Added Sugar**	2.2	2.5	1.2	2.3	2.1	1.2	2.8	2.3	1.9	2.5
**E% Other Carbohydrates**	51.6	48.5	50.4	51.1	50.3	45.2	51.0	53.3	50.3	50.5
**Total Energy intake (kCal)**	1,786 (489)	2,560 (335)	1,665 (387)	1,800 (360)	1,816 (337)	1,794 (390)	2,473 (421)	1,804 (456)	1,720 (350)	1,636 (357)
**Demographics**										
**Age (y)**	7.03 (6.52, 8.02)	7.33 (6.61, 8.22)	6.96 (6.44, 7.83)	6.87 (6.31, 7.32)	7.35 (6.87, 8.00)	7.32 (6.77, 8.12)	7.59 (6.95, 8.07)	7.27 (6.69, 7.90)	7.12 (6.56, 7.70)	6.90 (6.36, 7.52)
**Indigenous**										
No	8 (23%)	12 (17%)	29 (34%)	14 (24%)	22 (31%)	21 (28%)	7 (14%)	16 (21%)	13 (28%)	18 (21%)
Yes	27 (77%)	58 (83%)	56 (66%)	44 (76%)	50 (69%)	55 (72%)	42 (86%)	61 (79%)	34 (72%)	69 (79%)
**Caregiver Highest Education**										
High school or less	25 (71%)	42 (60%)	46 (54%)	33 (57%)	42 (58%)	47 (62%)	28 (57%)	39 (51%)	25 (53%)	51 (59%)
More than school	10 (29%)	28 (40%)	39 (46%)	25 (43%)	30 (42%)	29 (38%)	21 (43%)	38 (49%)	22 (47%)	36 (41%)
**Jurisdiction**										
Alaska	0 (0%)	7 (10%)	5 (5.9%)	5 (8.6%)	7 (9.7%)	8 (11%)	1 (2.0%)	2 (2.6%)	11 (23%)	6 (6.9%)
American Samoa	5 (14%)	23 (33%)	6 (7.1%)	7 (12%)	11 (15%)	6 (7.9%)	22 (45%)	11 (14%)	2 (4.3%)	9 (10%)
CNMI	5 (14%)	4 (5.7%)	31 (36%)	11 (19%)	19 (26%)	21 (28%)	8 (16%)	15 (19%)	10 (21%)	12 (14%)
FAS	10 (29%)	6 (8.6%)	16 (19%)	16 (28%)	11 (15%)	18 (24%)	4 (8.2%)	11 (14%)	15 (32%)	20 (23%)
Guam	7 (20%)	21 (30%)	16 (19%)	10 (17%)	14 (19%)	17 (22%)	8 (16%)	18 (23%)	6 (13%)	24 (28%)
Hawaii	8 (23%)	9 (13%)	11 (13%)	9 (16%)	10 (14%)	6 (7.9%)	6 (12%)	20 (26%)	3 (6.4%)	16 (18%)

All values are n (%) or mean (SD), or median (IRQ) if not normally distributed, indicated by [a]

Abbreviations: BMI z-score = body mass index z-score, MVPA = moderate-to-vigorous physical activity, CNMI = Commonwealth of the Northern Mariana Islands; FAS = Freely Associated State; E% = % of daily energy intake.

Alternative visualization of results.

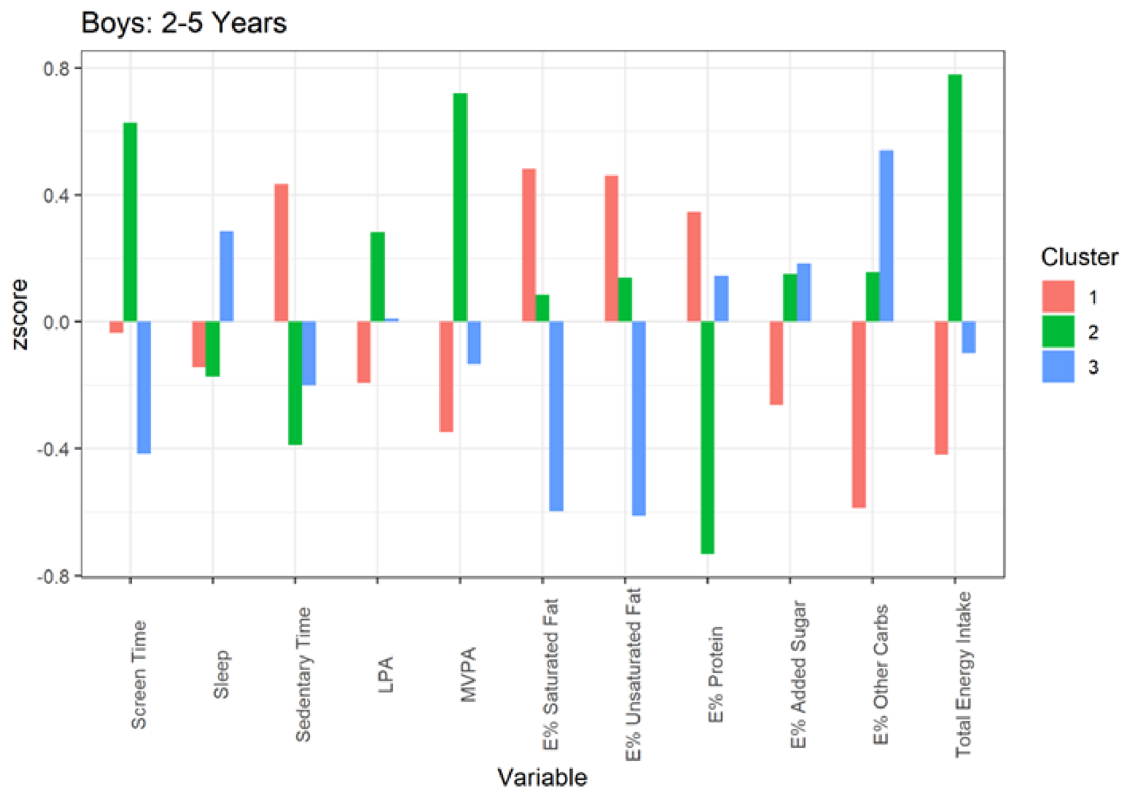

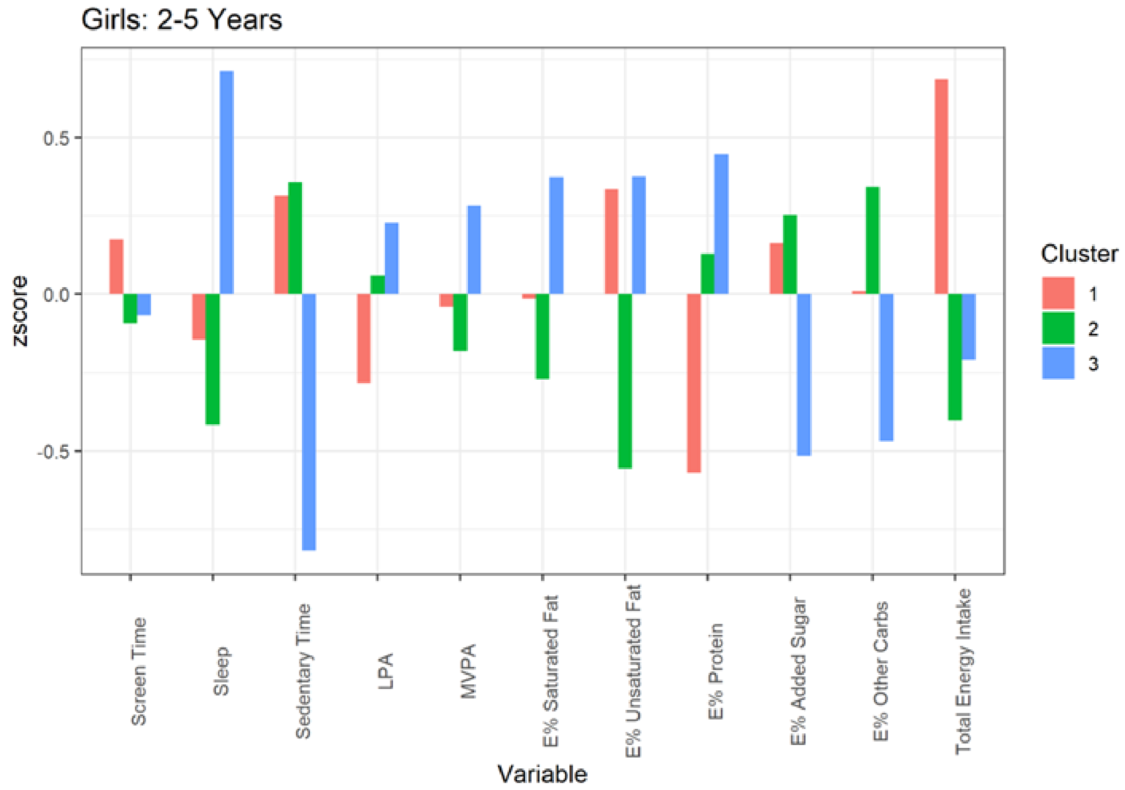

Figure S10. Variable View for Younger Children (2-5 years): z-scores relative to the grand mean.

LPA=Light Physical Activity, MVPA=Moderate-to-Vigorous Physical Activity, Carbs=Carbohydrates. E% represents % of total energy.

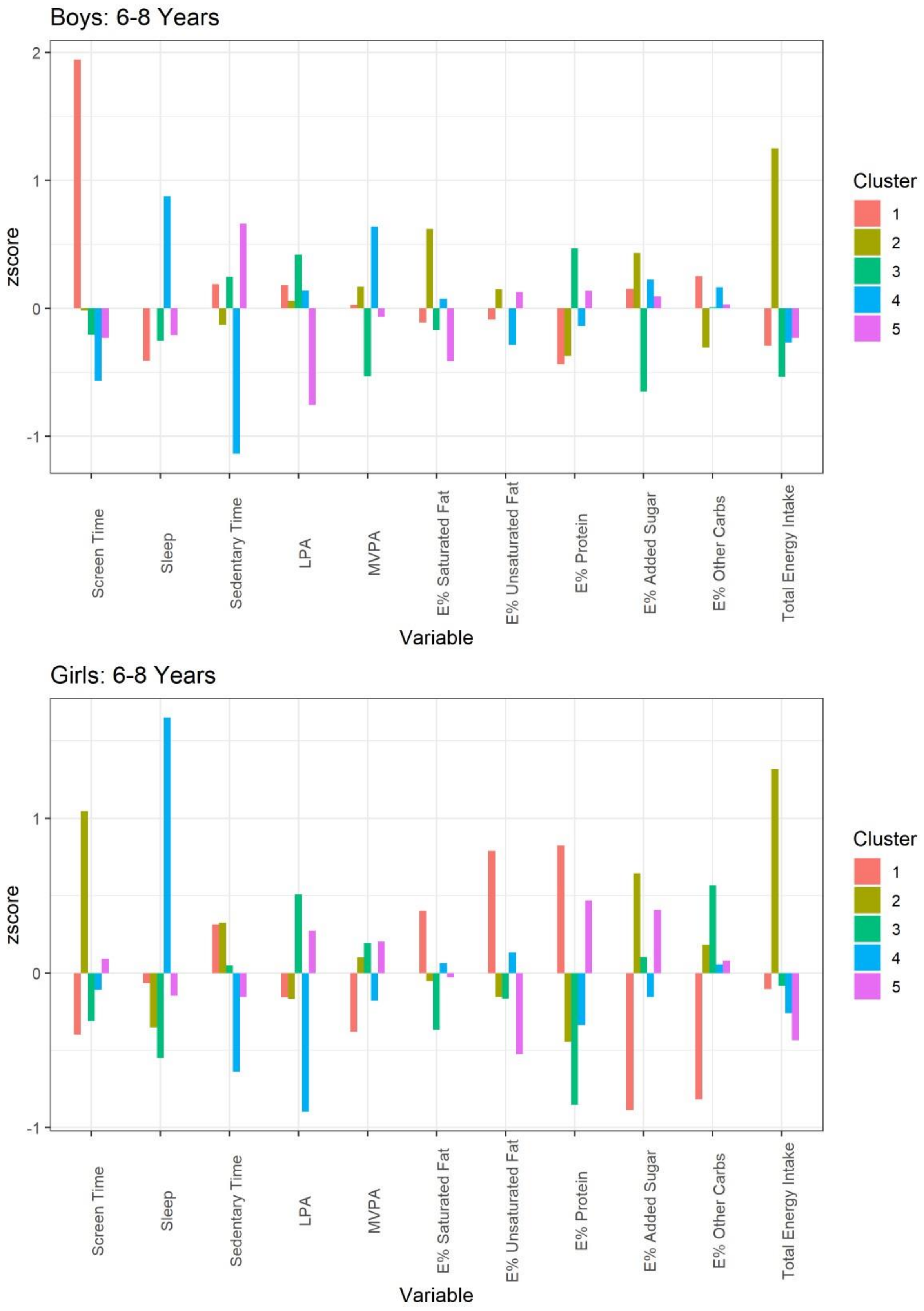

Figure S11. Variable View for Older Children (6-8 years): z-scores relative to the grand mean. LPA=Light Physical Activity, MVPA=Moderate-to-Vigorous Physical Activity, Carbs=Carbohydrates. E% represents % of total energy.

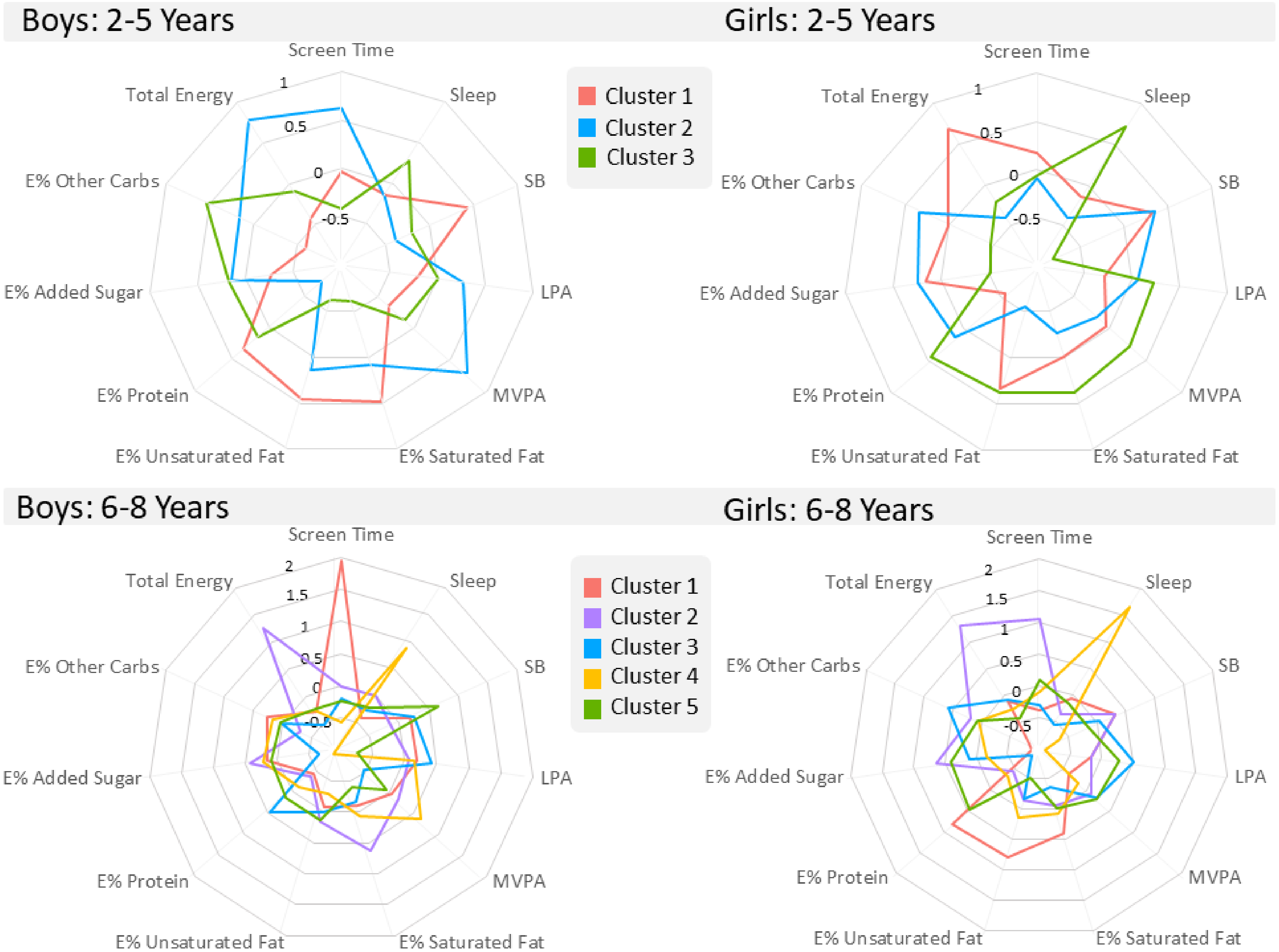

Figure S12. Cluster solutions by sex and age group: average lifestyle behavior variables (z-scores) across clusters. Abbreviations: SB=sedentary behavior (from accelerometry); LPA=light-intensity physical activity, MVPA=moderate-to vigorous intensity physical activity.

Table S4. Relationships between cluster membership and anthropometric measures

	Boys 2-5 years				Girls 2-5 years			
	**n**	**Mean [SE]**	**Beta [95% CI]**	**p**	**n**	**Mean [SE]**	**Beta [95% CI]**	**p**
BMI z-score	556			**0.04**	567			**0.005**
Cluster 1	218	0.62 [0.14]	Ref	-	180	0.51 [0.13]	Ref	-
Cluster 2	142	0.47 [0.15]	-0.15 [-0.39; 0.09]	0.21	221	0.43 [0.12]	-0.09 [-0.29; 0.11]	0.39
Cluster 3	196	0.33 [0.14]	-0.28 [-0.50; -0.07]	**0.01**	166	0.17 [0.12]	-0.34 [-0.55; -0.13]	**0.002**
Waist-to-Height (%)	537			**0.006**	551			0.21
Cluster 1	210	50.3 [0.26]	Ref	**-**	172	51.0 [0.51]		
Cluster 2	138	50.2 [0.32]	-1.09 [-1.89; -0.29]	**0.008**	219	51.0 [0.50]	-0.06 [-0.83; 0.71]	0.88
Cluster 3	189	50.2 [0.28]	-1.06 [-1.79; -0.33]	**0.005**	160	50.4 [0.51]	-0.66 [-1.48; 0.17]	0.12
	**Boys 6-8 years**				**Girls 6-8 years**			
	**n**	**Mean [SE]**	**Beta [95% CI]**	**p**	**n**	**Mean [SE]**	**Beta [95% CI]**	**p**
BMI z-score	320			**<0.001**	336			**0.01**
Cluster 1	35	1.08 [0.23]	Ref	-	76	0.64 [0.20]	Ref	-
Cluster 2	70	0.65 [0.20]	-0.43 [-0.87; 0.01]	0.05	49	0.06 [0.23]	-0.57 [-0.98; -0.17]	**0.006**
Cluster 3	85	0.65 [0.18]	-0.43 [-0.84; -0.02]	**0.04**	77	0.16 [0.20]	-0.48 [-0.81; -0.14]	**0.005**
Cluster 4	58	0.44 [0.20]	-0.64 [-1.08; -0.20]	**0.004**	47	0.29 [0.22]	-0.35 [-0.72; 0.03]	0.07
Cluster 5	72	0.15 [0.19]	-0.93 [-1.35; -0.51]	**<0.001**	87	0.14 [0.20]	-0.50 [-0.83; -0.18]	**0.003**
Waist-to-Height (%)	313			**<0.001**	327			**0.005**
Cluster 1	35	49.9 [0.95]	Ref	-	72	49.4 [0.77]	Ref	-
Cluster 2	70	48.1 [0.77]	-1.79 [-4.03; 0.45]	0.12	49	46.5 [0.94]	-2.95 [-4.98; -0.91]	**0.005**
Cluster 3	83	50.3 [0.65]	0.40 [-1.70; 2.51]	0.71	77	46.4 [0.76]	-3.03 [-4.72; -1.35]	**<0.001**
Cluster 4	56	47.6 [0.82]	-2.30 [-4.59; -0.02]	**0.048**	44	47.8 [0.92]	-1.67 [-3.62; 0.28]	0.09
Cluster 5	69	46.6 [0.73]	-3.32 [-5.51; -1.12]	**0.003**	85	46.9 [0.74]	-2.49 [-4.14; -0.85]	**0.003**

Note: all analyses (including Mean [SE] values presented above) were adjusted for child's age, indigenous status, parental education level and clustering at jurisdiction and community levels. The *p* values presented in the row corresponding to the obesity marker are for the global F test, while the remaining *p* values are for pair-wise comparisons with the reference group (Cluster 1). Statistically significant *p* values at an alpha of 0.05 are shown in bold.

Additional analyses of Healthy Eating Index across clusters.

This supplementary analysis compares the Healthy Eating Index (HEI-2020) component scores across the clusters. These variables were derived from the same dietary recalls described in the main paper. Dietary Reference Intake values were used to evaluate nutrient intake and meeting recommendations based on age and sex (1). The HEI-2020 scores reflect the 2020 – 2025 Dietary Guidelines for Americans with higher scores indicating better adherence to federal dietary guidelines (2). The HEI-2020 consists of 13 components that together sum a total maximum score of 100 points (total HEI) equating to optimal alignment of the guidelines. The 13 components can be divided into nine components focused on adequacy (Total Fruits, Whole Fruits, Total Vegetables, Greens and Beans, Whole Grains, Dairy, Total Protein Foods, Seafood and Plant Proteins, Fatty Acids) and four components focused on moderation (Refined Grains, Sodium, Saturated Fats, Added Sugars). All components except saturated fats and calories from solid fats, alcoholic beverages, and added sugars were calculated as per 1000 kcal, and 5, 10, or 20 points were assigned to optimal intakes. Saturated fats, sodium, and added sugars were reverse scored (2). The simple HEI scoring algorithm was used for calculating HEI-2020 scores of the dietary data for each day. The dietary intake amounts for the indices were then averaged across the two days, weighted for weekday and weekend days.

References

1. Meyers LD, Hellwig JP, Otten JJ. Dietary reference intakes: the essential guide to nutrient requirements: National Academies Press; 2006.

2. Shams-White MM, Pannucci TE, Lerman JL, Herrick KA, Zimmer M, Mathieu KM, et al. Healthy Eating Index-2020: Review and Update Process to Reflect the Dietary Guidelines for Americans, 2020-2025. Journal of the Academy of Nutrition and Dietetics. 2023.

Table S5. HEI-2020 Component scores across clusters: 2-5 years

Variable	Boys 2-5 years			Girls 2-5 years		
	**Cluster 1,** N = 218	**Cluster 2,** N = 142	**Cluster 3,** N = 196	**Cluster 1,** N = 180	**Cluster 2,** N = 221	**Cluster 3,** N = 166
**BMI z-score**	0.50 (1.17)	0.40 (1.17)	0.32 (1.05)	0.55 (0.96)	0.40 (1.02)	0.14 (1.02)
**Total Vegetables**	1.35 (1.82)	1.09 (1.63)	1.66 (1.88)	1.66 (2.10)	1.65 (2.03)	1.18 (2.12)
**Greens and Beans**	0.00 (0.02)	0.00 (0.00)	0.00 (0.62)	0.00 (0.06)	0.00 (0.25)	0.00 (0.09)
**Total Fruit**	2.50 (3.59)	2.77 (3.27)	3.53 (3.37)	3.03 (3.10)	3.47 (3.10)	2.50 (3.47)
**Whole Fruit**	2.50 (3.67)	3.57 (3.28)	3.70 (3.13)	3.59 (3.31)	3.57 (2.97)	2.90 (4.05)
**Whole Grains**	0.00 (3.21)	0.04 (2.12)	0.04 (2.82)	0.34 (2.64)	0.52 (2.95)	0.00 (2.56)
**Dairy**	5.90 (6.36)	4.21 (4.37)	5.75 (6.47)	5.16 (4.33)	6.77 (6.26)	5.60 (6.35)
**Total Protein**	5.00 (0.94)	4.68 (1.24)	4.83 (1.16)	4.73 (1.27)	4.53 (1.56)	5.00 (0.78)
**Seafood and Plant Protein**	1.87 (3.45)	1.78 (3.58)	2.62 (4.28)	1.64 (3.50)	2.50 (4.68)	2.50 (3.99)
**Fatty Acid Ratio**	3.35 (3.95)	4.04 (4.64)	3.90 (4.41)	5.26 (4.49)	2.95 (4.45)	3.89 (4.51)
**Sodium**	5.35 (4.41)	6.55 (4.06)	8.00 (4.14)	6.41 (4.51)	7.38 (4.54)	5.79 (5.02)
**Refined Grains**	3.95 (5.16)	2.87 (4.30)	3.02 (4.27)	3.24 (4.94)	3.35 (4.48)	4.21 (4.95)
**Saturated Fat**	4.79 (4.12)	5.87 (3.66)	8.28 (3.06)	6.97 (3.43)	7.80 (3.38)	5.66 (4.39)
**Added Sugar**	9.05 (2.22)	8.31 (3.58)	8.14 (3.41)	7.95 (2.77)	7.93 (2.80)	9.39 (2.14)
**HEI total**	47.77 (14.74)	46.61 (13.80)	53.15 (14.23)	50.07 (15.13)	50.45 (14.97)	49.30 (15.21)

BMI = body mass index, HEI = Healthy Eating Index-2020

Table S6. HEI-2020 Component scores across clusters: 6-8 years

Variable	Boys 6-8 y Cluster 1, N = 35	Cluster 2, N = 70	Cluster 3, N = 85	Cluster 4, N = 58	Cluster 5, N = 72	Girls 6-8 y Cluster 1, N = 76	Cluster 2, N = 49	Cluster 3, N = 77	Cluster 4, N = 47	Cluster 5, N = 87
**BMI z-score**	1.02 (0.88)	0.70 (1.00)	0.59 (1.23)	0.36 (1.16)	0.32 (1.10)	0.57 (1.15)	0.31 (1.14)	0.29 (1.06)	0.24 (1.06)	0.17 (1.10)
**Total Vegetables**	1.20 (2.27)	1.88 (1.26)	2.08 (2.39)	1.74 (1.98)	1.78 (2.17)	1.64 (2.08)	1.66 (1.38)	1.89 (2.18)	1.80 (2.46)	1.84 (2.31)
**Greens and Beans**	0.00 (0.20)	0.00 (1.34)	0.00 (0.44)	0.00 (1.02)	0.00 (0.14)	0.00 (0.07)	0.00 (0.00)	0.00 (1.43)	0.00 (0.00)	0.00 (0.21)
**Total Fruit**	2.88 (3.35)	2.80 (2.46)	2.79 (4.25)	2.82 (2.84)	3.09 (3.36)	3.02 (3.50)	2.67 (1.66)	4.21 (3.01)	3.31 (3.28)	2.91 (2.88)
**Whole Fruit**	3.30 (4.25)	3.66 (3.07)	2.70 (4.89)	3.18 (3.89)	3.57 (3.53)	3.57 (3.78)	3.57 (2.23)	3.88 (3.09)	3.46 (3.62)	3.56 (3.52)
**Whole Grains**	0.00 (1.15)	0.62 (2.68)	0.00 (2.20)	0.40 (2.73)	0.00 (2.78)	0.00 (2.11)	0.61 (2.40)	0.15 (2.52)	1.77 (4.63)	0.58 (3.65)
**Dairy**	4.04 (6.30)	5.63 (4.36)	4.66 (7.09)	5.89 (6.90)	4.92 (5.05)	3.49 (6.79)	6.59 (4.85)	4.64 (5.70)	5.03 (4.48)	6.40 (5.40)
**Total Protein**	4.81 (0.71)	4.92 (0.66)	5.00 (0.32)	4.88 (0.98)	5.00 (0.95)	5.00 (0.00)	4.66 (1.10)	4.43 (1.64)	4.70 (1.37)	5.00 (1.04)
**Seafood and Plant Protein**	1.43 (2.49)	2.50 (4.10)	1.84 (3.52)	2.56 (2.58)	2.50 (3.83)	2.50 (4.87)	2.16 (1.91)	2.50 (3.26)	1.43 (2.49)	2.49 (3.18)
**Fatty Acid Ratio**	3.62 (2.74)	3.13 (3.35)	4.07 (3.86)	2.86 (4.29)	5.57 (4.35)	5.08 (3.70)	3.74 (3.98)	5.48 (4.38)	4.15 (3.79)	2.86 (3.59)
**Sodium**	5.88 (4.94)	5.57 (4.44)	5.39 (5.46)	6.96 (3.97)	6.87 (5.33)	5.26 (4.27)	7.35 (2.93)	6.55 (4.34)	6.13 (4.79)	6.74 (4.21)
**Refined Grains**	2.49 (3.28)	5.11 (4.69)	2.20 (4.29)	2.81 (5.01)	2.85 (3.76)	4.39 (4.83)	3.91 (5.16)	2.75 (4.17)	3.95 (3.97)	4.52 (4.60)
**Saturated Fat**	7.14 (4.46)	4.87 (4.45)	7.34 (4.93)	6.17 (5.19)	7.66 (3.19)	5.24 (5.05)	5.80 (4.48)	7.65 (3.83)	5.82 (3.02)	6.67 (3.88)
**Added Sugar**	8.92 (3.42)	7.66 (2.88)	10.00 (1.10)	8.18 (2.59)	8.95 (3.23)	10.00 (0.60)	7.36 (4.38)	8.51 (2.81)	8.43 (1.99)	7.36 (3.69)
**HEI total**	46.96 (12.34)	48.12 (12.97)	47.18 (15.18)	47.79 (14.58)	51.55 (14.27)	49.59 (14.11)	48.96 (12.19)	50.00 (15.51)	48.94 (15.25)	49.98 (17.18)

BMI = body mass index, HEI = Healthy Eating Index-2020
